# Upregulation of CD11A on Hematopoietic Stem Cells Denotes the Loss of Long-Term Reconstitution Potential

**DOI:** 10.1016/j.stemcr.2014.09.007

**Published:** 2014-10-16

**Authors:** John W. Fathman, Nathaniel B. Fernhoff, Jun Seita, Connie Chao, Vanessa M. Scarfone, Irving L. Weissman, Matthew A. Inlay

**Affiliations:** 1 Stanford Institute for Stem Cell Biology and Regenerative Medicine, Stanford University, Stanford, CA 94305, USA; 2 Sue and Bill Gross Stem Cell Research Center, Department of Molecular Biology and Biochemistry, University of California Irvine, Irvine, CA 92697, USA; 3 Ludwig Center for Cancer Stem Cell Research and Medicine, Stanford University, Stanford, CA 94305, USA

## Abstract

Small numbers of hematopoietic stem cells (HSCs) generate large numbers of mature effector cells through the successive amplification of transiently proliferating progenitor cells. HSCs and their downstream progenitors have been extensively characterized based on their cell-surface phenotype and functional activities during transplantation assays. These cells dynamically lose and acquire specific sets of surface markers during differentiation, leading to the identification of markers that allow for more refined separation of HSCs from early hematopoietic progenitors. Here, we describe a marker, CD11A, which allows for the enhanced purification of mouse HSCs. We show through in vivo transplantations that upregulation of CD11A on HSCs denotes the loss of their long-term reconstitution potential. Surprisingly, nearly half of phenotypic HSCs (defined as Lin^−^KIT^+^SCA-1^+^CD150^+^CD34^−^) are CD11A^+^ and lack long-term self-renewal potential. We propose that CD11A^+^Lin^−^KIT^+^SCA-1^+^CD150^+^CD34^−^ cells are multipotent progenitors and CD11A^−^Lin^−^KIT^+^SCA-1^+^CD150^+^CD34^−^ cells are true HSCs.

## Highlights

• CD11A separates phenotypic HSCs (Lin^−^KIT^+^SCA-1^+^CD150^+^CD34^−^) into two fractions
• All long-term reconstitution activity is within the CD11A^−^ fraction of bone marrow
• CD11A blocking antibodies do not affect BM homing or long-term engraftment of HSCs

## Teaser

In this study, Inlay, Fathman and colleagues identified a mouse marker, CD11A, that separated hematopoietic stem cells (HSCs, defined as Lin^−^KIT^+^SCA-1^+^ CD150^+^ CD34^−^) into two distinct populations. Only CD11A^−^ HSCs were able to engraft long-term upon transplantation and give rise to all blood cell types, indicating that only the CD11A^−^ fraction of phenotypic HSCs are true functional HSCs.

## Introduction

Since their identification and isolation over 25 years ago (Spangrude et al., 1988), hematopoietic stem cells (HSCs) have arguably become the most well-characterized tissue-specific “adult” stem cell. HSCs reside atop the hematopoietic hierarchy and give rise to functional effector cells through a succession of increasingly committed downstream progenitor cell stages (Seita and Weissman, 2010). Our understanding of the molecular basis for lineage determination and self-renewal has depended critically on our ability to identify and isolate HSCs and their downstream progeny with high purity. HSCs are primarily quiescent, but their immediate downstream progeny, multipotent progenitors (MPPs), are transit-amplifying cells and rapidly proliferate and differentiate to replenish the blood supply. Thus, reliably separating HSCs from MPPs is key to characterizing their distinct self-renewal and differentiation potentials, and considerable attention has been paid to markers that can better separate these populations, which include SCA-1, KIT, CD34, and CD150 (Kiel et al., 2005). Analyses of purified HSCs transplanted into lethally irradiated mice at low numbers (1 to 50 cells per mouse) have revealed functional heterogeneity within phenotypic HSCs (Beerman et al., 2010; Benz et al., 2012; Lu et al., 2011). Beerman et al. demonstrated that higher levels of CD150 (SLAMF1) marked HSCs that are skewed toward myeloid cell fates, compared to CD150^int^ HSCs, which display a more balanced lineage output (Beerman et al., 2010). Other groups have shown heterogeneity of HSCs using a variety of markers such as cytokine receptors, other Slam family members, and adhesion molecules (Arai et al., 2004; Kiel et al., 2005; Wagers et al., 2002). Thus, even with the existing panel of markers, the HSC population is likely heterogeneous.

Based on our own gene expression analyses of HSCs and downstream progenitors (Seita and Weissman, 2010), we identified integrin alpha L (CD11A, *Itgal*) as a possible marker to better purify HSCs. CD11A heterodimerizes with CD18 (integrin beta-2) to form the adhesion molecule LFA-1 (lymphocyte function-associated anigten-1) (Cornwell et al., 1993). LFA-1 is expressed on all leukocytes and plays important roles in many immunological processes, including transendothelial migration toward sites of inflammation (Van Epps et al., 1989), lymphocyte costimulation and effector-target cell interactions (Davis et al., 1999), and formation of the T cell immunological synapse (Grakoui et al., 1999).

In this study, we show that CD11A has bimodal expression on phenotypic HSCs (Lin^−^KIT^+^SCA-1^+^FLK2^−^CD150^+^CD34^−^). Our data show that the CD11A^−^ fraction of HSCs contains all functional HSC activity, with the CD11A^+^ fraction composed of more differentiated cells that lack long-term self-renewal activity.

## Results and Discussion

### Bimodal Expression of CD11A on Phenotypic HSCs in Mice

**Figure 1:**
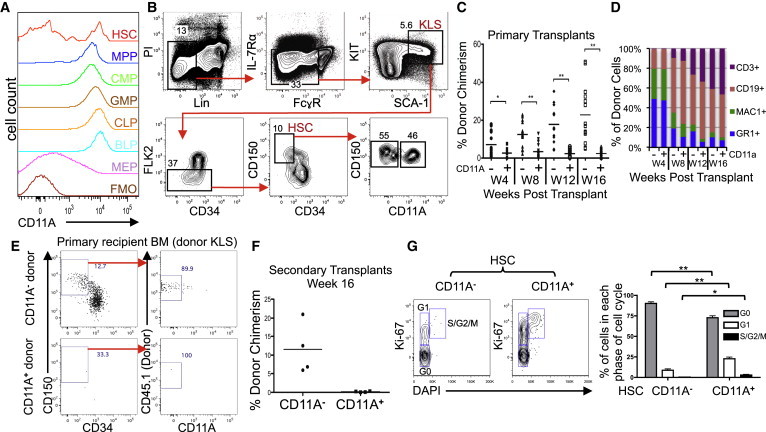
Bimodal Expression of CD11A on Phenotypic HSCs (A) BM populations were analyzed for cell-surface expression of CD11A. Fluorescence minus one (FMO) was used as the negative control, gated on Lin^−^ cells. (B) Gating scheme of murine HSCs. Phenotypic HSCs are gated on live cells (PI^−^), Lin^−^ (CD3ε^−^, CD19^−^, NK1.1^−^, GR1^−^, TER119^−^), IL-7Rα^−^, FcγR^lo^, KIT^+^, SCA-1^+^, FLK2^−^, CD150^+^, and CD34^−^. The markers IL-7Rα and FcγR are typically not necessary to identify HSCs but are shown here for additional resolution. (C) CD11A^−^ (−) and CD11A^+^ (+) HSC subfractions (50 cells/mouse) were transplanted into five lethally irradiated congenic recipients along with 2 × 10^5^ competitive BM cells. Mice were analyzed at 4 (W4), 8 (W8), 12 (W12), and 16 (W16) weeks after transplantation for total donor blood chimerism (^∗^p < 0.01, ^∗∗^p < 0.0001). Graph includes data from three independent experiments. (D) Donor-derived lineage distribution for granulocytes (MAC1^+^GR1^+^, “GR1,” blue), macrophages (MAC1^+^GR1^−^, “MAC1,” green), B cells (CD19^+^, red), and T cells (CD3ε^+^, purple) from CD11A^−^ (−) and CD11A^+^ (+) HSC subfractions. (E) BM from primary recipient mice transplanted with the CD11A^−^ and CD11A^+^ HSC subfractions were analyzed for CD11A expression in donor-derived HSC. Only donor-derived KLS cells (Lin^−^KIT^+^SCA-1^+^) are shown. (F) BM from primary recipients transplanted with CD11A^−^ and CD11A^+^ HSC subfractions were harvested at 16 weeks posttransplant, and 2 × 10^5^ cells were retransplanted into lethally irradiated congenic secondary recipients. Blood was analyzed 16 weeks posttransplant for long-term engraftment. (G) Cell-cycle analysis of CD11A^−^ and CD11A^+^ HSC subfractions. BM was stained with Ki-67 and DAPI to identify the percentage of CD11A^−^ and CD11A^+^ HSCs in G0 (Ki-67^−^ DAPI^−^), G1 (Ki-67^+^ DAPI^−^) and S/G2/M (Ki-67^+^ DAPI^+^) phases. Statistics are Student’s unpaired t test (n = 4, ^∗^p < 0.05, ^∗∗^p < 0.01).

Based on a screen of a microarray database spanning over 35 mouse hematopoietic populations (Seita and Weissman, 2010), we discovered that HSCs express much lower levels of CD11A than downstream progenitors (Figures S1A and S1B available online). We examined mouse whole bone marrow (BM) with anti-CD11A antibodies (Abs) to measure CD11A surface expression by flow cytometry (Figure 1A). All mature lymphocytes were positive for CD11A on their cell surface (data not shown), and almost all hematopoietic progenitor populations expressed high levels of CD11A, including MPPs and both myeloid (CMP, GMP) and lymphoid (CLP, BLP) committed progenitors (Figure 1A, see Supplemental Experimental Procedures for definitions and surface marker phenotypes). Only the megakaryocyte/erythrocyte progenitor (MEP) expressed low levels of CD11A. In contrast, HSCs (defined as Lin^−^KIT^+^SCA-1^+^FLK2^−^ CD150^+^CD34^−^) had a bimodal expression of CD11A (Figures 1A and 1B). The CD11A^−^ fraction accounts for anywhere from 30%–70% of the phenotypic HSC population, depending on the strain and age of the mouse (Figure 1B).

### CD11A^−^ Fraction Enriches for HSC Activity

The gold standard assay to functionally identify HSCs is long-term multilineage reconstitution after intravenous transplantation into lethally irradiated mice. We transplanted 50 cells from the phenotypic HSC CD11A^−^ and CD11A^+^ subpopulations into lethally irradiated congenic recipients and analyzed the blood for total donor chimerism and lineage distribution every 4 weeks (Figures 1C and 1D). At all time points, the CD11A^−^ fraction gave a higher burst size compared to CD11A^+^ fraction (Figure 1C). This trend magnified over the 16 week time course, with the contribution of the CD11A^−^ fraction increasing over time. Conversely, the median chimerism from the CD11A^+^ fraction decreased overall during this span. We also examined the lineage distribution of donor cells in the recipient blood and observed no difference in lineage bias between the CD11A^−^ and CD11A^+^ subfractions of HSCs (Figure 1D).

Based on the phenotypic similarities between the two subpopulations and the similarity in lineage potentials, we hypothesized that the CD11A^−^ fraction is upstream of, and gives rise to, the CD11A^+^ fraction. In support of this notion, the CD11A^−^ HSC subfraction could give rise to both CD11A^−^ and CD11A^+^ HSC subtypes in the BM of primary recipients, whereas we saw no phenotypic HSCs in the donor BM population from the CD11A^+^ fraction transplants (Figure 1E).

The most definitive test of self-renewal capability is demonstrated in secondary transplants. We therefore isolated BM from the primary recipients of CD11A^−^ and CD11A^+^ transplants and retransplanted them into secondary hosts (Figure 1F). When we analyzed the secondary hosts 16 weeks after transplant, we only detected donor-derived cells from the CD11A^−^ HSC fraction, proving they have functional self-renewal activity.

HSCs, which are mainly quiescent, likely have significant differences in the expression of cell-cycle regulators compared to the more robustly proliferative MPPs. We compared gene expression microarrays of these HSC subpopulations to existing arrays of MPPs and downstream progenitors for expression of cell-cycle genes (Figure S1C). Our data indicate that among all populations, the CD11A^−^ HSC subfraction had the lowest expression of key cell-cycle regulators, including cyclins and cyclin-dependent kinases. The CD11A^+^ fraction appeared to upregulate many of these cell-cycle promoters, expressing levels between that of CD11A^−^ HSCs and downstream multipotent progenitors. In addition, we examined the cell-cycle status of CD11A^−^ and CD11A^+^ fractions of HSCs and determined that the CD11A^−^ fraction had significantly greater frequency of cells in G0, and significantly fewer in G1 and S/G2/M phase (Figure 1G).

When examining FMO (fluorescence minus one) controls, it did not appear that CD11A was completely unexpressed in the CD11A^−^ fraction of HSCs (Figure S2A), making it difficult to gate these HSC subfractions based on the FMO. However, by comparing CD11A expression to other HSC markers, such as CD34, FLK2, and CD150, a clear population can be identified (Figure S2B). We also compared CD11A expression on HSCs to two new HSC markers EPCR (*Procr*, CD201) and CD9 (Balazs et al., 2006; Karlsson et al., 2013) and found that CD11A was able to identify a population that would not be separable using either EPCR or CD9 (Figure S2C). However, we did find that EPCR in combination with CD11A could clearly identify a subset of EPCR^+^ CD11A^−^ HSCs (Figure S2D). In fact, EPCR and CD11A alone could highly enrich for HSCs from whole BM, at approximately 40% purity using all other HSC markers (Figure S2E). If BM is first enriched for KIT^+^ cells using anti-KIT microbeads, the purity of HSCs improves to 74% within the EPCR^+^ CD11A^−^ fraction (Figure S2E).

### All HSCs Are within the CD11A^−^ Fraction of BM

**Figure 2:**
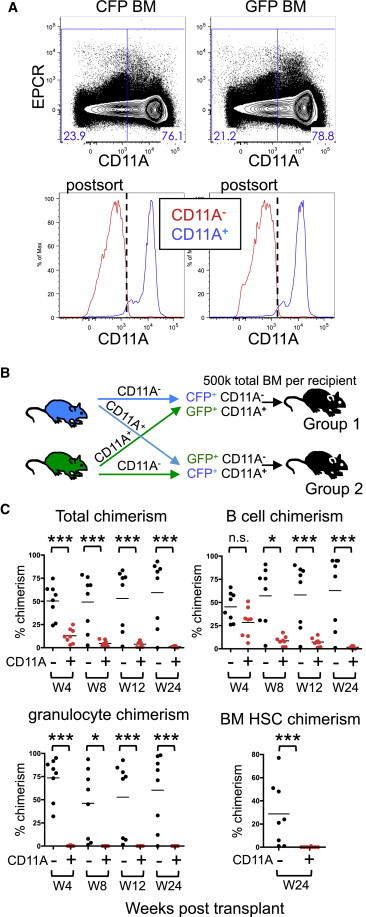
CD11A Antibody Separates BM Reconstituting Activity (A) CFP^+^ and GFP^+^ BM cells were sorted based on CD11A expression into CD11A^−^ (red) and CD11A^+^ (blue) fractions. (B) Recipient mice were transplanted with CD11A^−^ and CD11A^+^ fractions in two groups. Group 1 received CFP^+^ CD11A^−^ and GFP^+^ CD11A^+^ cells. Group 2 received GFP^+^ CD11A^−^ and CFP^+^ CD11A^+^ cells. All mice received a total of 500,000 cells at the physiologic ratio of CD11A^−^ and CD11A^+^ BM cells. (C) Time-course analysis of donor chimerism. Donor chimerism derived from CD11A^−^ and CD11A^+^ BM cells is indicated for total cells (upper left), B cells (upper right), and granulocytes (lower left) in the peripheral blood at 4, 8, 12, and 24 weeks posttransplant, and HSCs (lower right) in the BM at 24 weeks. Data from groups 1 and 2 are pooled, and chimerism from CD11A^−^ fraction (−, black circles) is shown on the left, and CD11A^+^ fraction (+, red circles) is shown on the right at each time point. Percentages are out of total cells, including those of the host. ^∗^p < 0.05, ^∗∗∗^p < 0.001 (Student’s unpaired t test).

It is possible that, because of the high number of markers and stringent sorting criteria we used to purify HSCs, we may have missed functional HSCs that fall outside of our gates that may be CD11A^+^. To determine whether any long-term reconstituting activity exists within CD11A^+^ BM cells, we sorted whole BM based only on CD11A expression into positive and negative fractions and transplanted the entirety of each fraction into recipient mice (Figures 2A–2C). We designed our transplants to be competitive, sorting CD11A^−^ cells from GFP^+^ BM and CD11A^+^ cells from CFP^+^ BM (and vice versa) and then cotransplanting them into the same recipients (Figure 2B). At 4, 8, 12, and 24 weeks posttransplant, we identified donor cells from both CD11A^−^ and CD11A^+^ fractions in the recipient peripheral blood (Figure 2C). However, donor granulocytes were only from the CD11A^−^ fraction. Because granulocytes are short lived, they are a better indicator of HSC engraftment than longer-lived cells such as lymphocytes. Furthermore, when we examined the BM of recipient animals at 24-weeks, only CD11A^−^ BM gave rise to donor HSCs (Figure 2C). Our data clearly indicate that all HSCs reside within the CD11A^−^ fraction of BM.

### Anti-CD11A Antibody Does Not Inhibit Engraftment of HSCs or Homing of CD11A^+^ Cells into the BM

**Figure 3:**
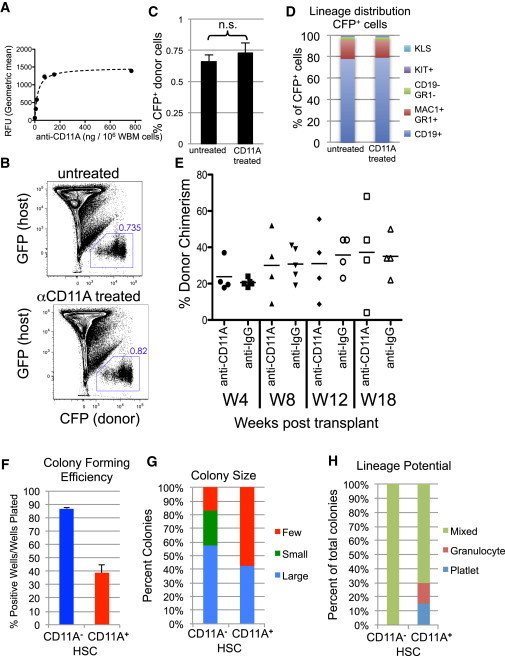
CD11A Antibody Does Not Inhibit BM Homing or Long-Term Engraftment (A) Saturation curve for anti-CD11A Ab on BM cells. (B and C) CFP^+^ bone marrow cells (10 × 10^6^/mouse) were untreated or stained with CD11A Ab and then transplanted into GFP^+^ unirradiated recipients (n = 5 each group). Three hours after transplantation, bones were harvested and analyzed for homing of CFP^+^ donor cells. (B) Representative BM from GFP hosts transplanted with untreated (top) and CD11A-Ab-treated (bottom) CFP^+^ BM. Live cells are shown. (C) Comparison of the percentage of CFP^+^ donor cells from untreated or CD11A-Ab-treated BM. Error bars are SD (n = 5) and are not significant (n.s.). (D) Lineage distribution of CFP^+^ donor-BM from untreated (left) and CD11A-Ab-treated (right) BM. The percentage of each lineage among donor-derived cells is shown. “KLS” is KIT^+^Lin^−^SCA-1^+^. (E) Phenotypic HSCs (Lin^−^KIT^+^SCA-1^+^FLK2^−^ CD150^+^CD34^−^) were purified and split into two groups, one was treated with anti-CD11A, and the other was treated with Rat anti-IgG isotype control. One hundred HSCs from each group were transplanted into four lethally irradiated congenic mice along with 2 × 10^5^ competitive BM cells. Mice were analyzed every 4 weeks for blood donor chimerism levels up to 4 months. Bars indicate average percentage of donor chimerism. Data are representative of two experiments. (F) Single-cell liquid cultures of CD11A^−^ (blue, left) and CD11A^+^ (red, right) HSC subfractions. The percentage of wells with colonies at day 12 is shown. Error bars are SD (n = 3 experiments). (G) The size of colonies at day 12 (large >1,000 cells, small <1,000 cells, few less than ten cells). (H) Distribution of the lineages produced by CD11A^−^ and CD11A^+^ subpopulations in vitro. For in vitro assays, each subpopulation was clone sorted into one 96-well plate. Data are representative of three independent experiments.

LFA-1 (CD11A/CD18) binds to ICAMs on the vascular lumen to help mediate extravasation (Van Epps et al., 1989). HSCs injected intravenously must migrate from the blood into the BM to engraft. It is known that the CD11A Ab clone we used can block LFA-1 binding to ICAMs (Weitz-Schmidt et al., 2001). Therefore, we next determined if blocking CD11A function with the CD11A Ab inhibited migration into the bone marrow and caused the low engraftment rates in the CD11A^+^ fraction we observed by transplantation. We first tested whether the CD11A Ab could block short-term homing of CD11A^+^ cells to the BM (Figures 3A–3D). We determined that 100 ng of CD11A Ab was sufficient to saturate 10^6^ BM cells (Figure 3A). We then harvested BM from CFP^+^ mice and stained them with CD11A Ab (with 100 ng/10^6^ cells), or left them untreated. We intravenously transplanted 10 million cells per recipient into unirradiated GFP^+^ mice and then analyzed the BM 3 hr posttransplant for donor (CFP^+^) cells. We found no significant difference in the percent of CFP^+^ donor cells in the bones between untreated and CD11A Ab-treated BM (Figures 3B and 3C). The distribution of donor lineages that homed to the BM was also equivalent, with the majority of homed cells being granulocytes and B cells, both of which express high levels of CD11A (Figure 3D). Thus, the CD11A Ab does not appear to block immediate homing to the BM of CD11A^+^ cells.

We next tested whether long-term engraftment was inhibited by CD11A Ab. We purified HSCs without the use of the CD11A Ab and then split the purified HSCs into two groups: one was treated with anti-CD11A Ab and the other with an isotype control (Figure 3E). We then transplanted 100 HSCs from each group and analyzed blood every 4 weeks for donor chimerism. At no point did we observe a statistical difference in donor chimerism between the two groups, demonstrating that the CD11A Ab did not inhibit engraftment, and that the inability of the CD11A^+^ fraction to engraft was not due to blocking LFA-1/ICAM interactions (Figure 3E).

To test the robustness of CD11A^−^ and CD11A^+^ HSC fractions’ regenerative capacity in the absence of transplantation, we used in vitro culture methods to assess the colony formation efficiency of the two populations, thus bypassing any homing or engraftment issues. Single cells were sorted directly into wells with media and cytokines (SCF, Flt3L, interleukin-3 [IL-3], TPO, EPO) and cultured for up to 12 days. The CD11A^−^ subpopulation displayed greater colony size (Figure 3F), colony forming efficiency (Figure 3G), and lineage potential (Figure 3H) than the CD11A^+^ subpopulation.

### HSCs Upregulate CD11A Expression during Granulocyte Colony-Stimulating Factor Mobilization

Administration of growth factors can activate HSCs and cause them to mobilize into the blood stream. Previous studies in mice have shown that mobilized HSCs in the periphery have elevated levels of several adhesion molecules (Wagers et al., 2002), and that pretreatment with anti-LFA-1 Abs inhibited the migration of HSCs out of the BM following IL-8-induced mobilization (Pruijt et al., 1998). We hypothesized that CD11A would be an important integrin in HSC mobilization and examined CD11A expression on BM and splenic HSCs following granulocyte colony-stimulating factor (G-CSF) mobilization (Figure S3). Indeed, CD11A increased as HSCs proliferated in the BM, peaking at day 3, just prior to HSC migration into the blood (Figure S3B). Moreover, HSCs in the spleen at day 5 showed similarly high CD11A expression as the BM HSCs did on day 3 (Figure S3D) (Morrison et al., 1997; Wright et al., 2001). Compared to the steady-state BM, where CD11A expression appears to mark the loss of self-renewal potential of HSCs, in the context of mobilization, it seems that HSCs upregulate CD11A in order to exit from the niche but still retain their reconstitution capacity.

### Frequency of the CD11A^−^ Subfraction of HSCs Increases with Age

Although HSC function declines with age, the frequency of phenotypic HSCs increases (Rossi et al., 2005). We next compared CD11A expression in middle-aged (12 months) and old mice (35 months), to young adult mice (3 months) (Figure S4). Although all progenitor populations except MEP appeared to uniformly express CD11A regardless of age, we found that within the HSC population, the fraction of CD11A^−^ cells increased with age (Figures S4A and S4C). At 35 months, almost all HSCs (>95%) are CD11A^−^. This correlates with the increase in phenotypic HSCs observed in aged mice (Figure S4B).

### Both CD11A^−^ and CD11A^+^ HSC Subpopulations from e17.5 Fetuses Can Engraft Long Term

**Figure 4:**
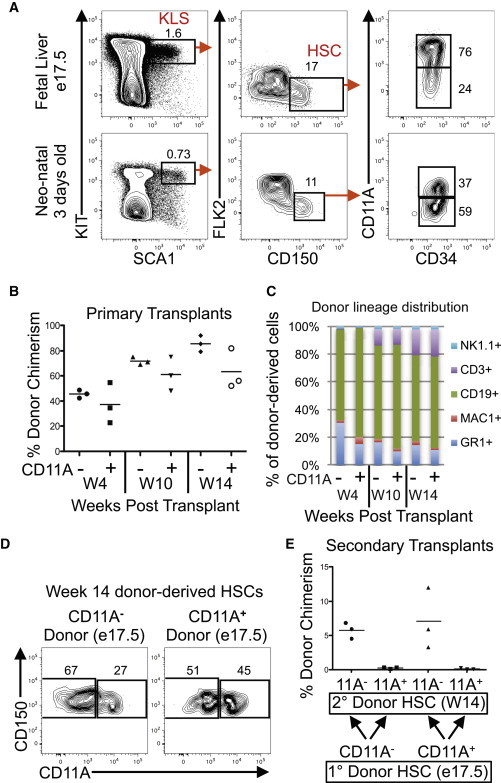
CD11A Expression on Fetal Liver and Neonate HSCs (A) HSC stains of e17.5 fetal liver (top row) and 3-day-old neonates (bottom row). Gates and percentages of KLS, HSC, and CD11A^−^ and CD11A^+^ subpopulations are shown. (B) Percentage of donor chimerism of CD11A^−^ and CD11A^+^ HSC subfractions sorted from e17.5 FL 4, 10, and 14 weeks after transplant. Bars indicate mean chimerism (n = 3). (C) Lineage distribution of donor-derived cells 4, 10, and 14 weeks after transplantation. (D) CD11A^−^ and CD11A^+^ donor-derived HSC subfractions from recipient mice 14 weeks after transplantation of CD11A^−^ (left) and CD11A^+^ (right) HSC subfractions from e17.5 FL. (E) Percentage of donor chimerism from secondary transplants of CD11A^−^ and CD11A^+^ donor-derived HSC subfractions reharvested from week 14 recipient mice. The two left columns are from the CD11A^−^ HSC subpopulation obtained from e17.5 FL, and the two right columns are from the CD11A^+^ HSC subpopulation obtained from e17.5 FL.

If the frequency of the CD11A^−^ fraction increased with age, we hypothesized that younger mice would have a decreased fraction of CD11A^−^ HSCs. We examined HSCs from 3-day-old neonates, as well as from embryonic day 17.5 (e17.5) fetal liver (FL), which is a site of embryonic hematopoiesis, and found that the fraction of CD11A^+^ phenotypic HSC was greater in e17.5 FL than in neonates or adult mice (Figure 4A). We next sorted and transplanted CD11A^−^ and CD11A^+^ subfractions of phenotypic HSCs from e17.5 FL (Figure 4B). Because the engraftability of embryonic HSCs is less than that from adults, we transplanted 450 cells of each. Surprisingly, we observed long-term donor chimerism from both CD11A^−^ and CD11A^+^ HSC subfractions (Figure 4B), with no differences in lineage distribution (Figure 4C). When we examined the recipients’ BM 14 weeks after transplantation, we found that both CD11A^−^ and CD11A^+^ donor e17.5 HSCs could give rise to both CD11A^−^ and CD11A^+^ HSCs in the recipients’ BM (Figure 4D). This indicates that at e17.5, functional HSCs are found within both CD11A^−^ and CD11A^+^ fractions. However, we then resorted donor-derived CD11A^−^ and CD11A^+^ phenotypic HSCs from the primary recipients for secondary transplants and found that within the secondary recipients, only the CD11A^−^ fraction engrafted, regardless of whether they originally came from CD11A^−^ or CD11A^+^ e17.5 HSCs (Figure 4E). Our data suggest that during embryonic development, long-term engraftable HSCs can come from either the CD11A^−^ or CD11A^+^ fraction, but, once engrafted in the adult bone marrow, only the CD11A^−^ fraction retains engraftability. In a related study, we show that all clonal multilineage potential in the embryo from e9.5 to e11.5 is contained within a similar CD11A^−^ population (Inlay et al., 2014). However, at e12.5 we detected multilineage potential from both CD11A^−^ and CD11A^+^ cells, consistent with our results here that engraftable HSCs are present in both the CD11A^−^ and CD11A^+^ fractions of HSCs in the embryo. In the embryo, hematopoiesis transitions through multiple sites including the yolk sac, dorsal aorta, fetal liver, and bone marrow (Christensen et al., 2004), and we hypothesize that CD11A may play an important role in the migration of embryonic HSCs and/or their precursors through each site.

In this study, we have discovered that only half of mouse phenotypic HSCs, as defined by the most stringent criteria (Lin^−^KIT^+^SCA-1^+^FLK2^−^CD150^+^CD34^−^), express CD11A, and that only the CD11A^−^ fraction of HSCs possesses the property of long-term multilineage differentiation and self-renewal upon intravenous transplantation into adult mice. As such, our data indicate that nearly half of the cells currently isolated as HSCs are not functional HSCs. This finding has clear implications for how we molecularly characterize HSCs and whether we can identify with certainty HSC niches in mouse bone marrow.

## Experimental Procedures

### Animals

All animal procedures were approved by the International Animal Care and Use Committee (IACUC) and the Stanford Administrative Panel on Laboratory Animal Care (APLAC). We used C57Bl/Ka-THY1.2 CD45.2 (B/Ka), C57Bl/Ka-THY1.1 CD45.2 (BA), and C57Bl/6-THY1.2 CD45.1 (CD45.1). Strains were derived and maintained in the I.L.W.’s laboratory.

### Antibodies

All antibodies, including clones and conjugations, used in this study are listed in Table S1.

### Cell Sorting

Bone marrow was harvested from donor mice by crushing bones and removing debris on a density gradient using Histopaque 1077 (Sigma). Where indicated, bone marrow was KIT enriched using anti-KIT (CD117) microbeads on an AutoMACS (Miltenyi Biotec). Cells were stained with Abs listed in Table S1 in PBS with 2% FCS. All cells were double sorted on a BD FACS-Aria II (Becton Dickinson). FlowJo software (Tree Star) was used for data analysis. Cells were sorted into ice-cold PBS with 2% FCS, or into tissue culture medium, or TRIzol (Invitrogen). For the cell-cycle analysis, KIT-enriched BM was stained with Abs for surface markers, fixed in 4% paraformaldehyde, and permeabilized with BioLegend Perm/Wash Buffer according to the manufacturer’s instructions. Cells were then stained with anti-Ki-67 (BioLegend), washed, and incubated with 1 μM DAPI for 10 min prior to analysis on a BD FACS Aria-II.

### Transplantation

Fifty to 450 HSCs (as indicated) were transplanted by retro-orbital injection into isofluorane-anesthetized recipients that had been lethally irradiated (900 rads, single dose) alongside 2 × 10^5^ helper bone marrow from congenically distinguishable wild-type mice. Blood was obtained from the tail vein of transplanted mice at various time points, and erythrocytes were sedimented using 2% dextran in PBS at 37°C for 30 min. Cells were stained with lineage Abs and analyzed on the BD FACSAria-II.

## Author Contributions

Experiments were designed by J.W.F. and M.A.I., advised by I.L.W., and performed by J.W.F., M.A.I., N.B.F., C.C., and V.M.S. J.S. contributed bioinformatics support and unpublished data. J.W.F. and M.A.I. wrote the manuscript, and I.L.W. edited it. Cell-cycle analysis was performed in the laboratory of M.A.I. All other experiments were performed in the laboratory of I.L.W.

## Accession Numbers

The GEO accession numbers for the microarrays of the CD11A^−^ HSC population are GSM1483039, GSM1483040, and GSM148041 and for the CD11A^+^ HSC population are GSM1483042 and GSM1483043 and are contained in experiment GSE34723.

## Supplemental Information

Document S1. Supplemental Experimental Procedures, Figures S1–S4, and Table S1

Document S2. Article plus Supplemental Information

## References

[bib1] Arai F., Hirao A., Ohmura M., Sato H., Matsuoka S., Takubo K., Ito K., Koh G.Y., Suda T. (2004). Tie2/angiopoietin-1 signaling regulates hematopoietic stem cell quiescence in the bone marrow niche. Cell.

[bib2] Balazs A.B., Fabian A.J., Esmon C.T., Mulligan R.C. (2006). Endothelial protein C receptor (CD201) explicitly identifies hematopoietic stem cells in murine bone marrow. Blood.

[bib3] Beerman I., Bhattacharya D., Zandi S., Sigvardsson M., Weissman I.L., Bryder D., Rossi D.J. (2010). Functionally distinct hematopoietic stem cells modulate hematopoietic lineage potential during aging by a mechanism of clonal expansion. Proc. Natl. Acad. Sci. USA.

[bib4] Benz C., Copley M.R., Kent D.G., Wohrer S., Cortes A., Aghaeepour N., Ma E., Mader H., Rowe K., Day C. (2012). Hematopoietic stem cell subtypes expand differentially during development and display distinct lymphopoietic programs. Cell Stem Cell.

[bib5] Christensen J.L., Wright D.E., Wagers A.J., Weissman I.L. (2004). Circulation and chemotaxis of fetal hematopoietic stem cells. PLoS Biol..

[bib6] Cornwell R.D., Gollahon K.A., Hickstein D.D. (1993). Description of the leukocyte function-associated antigen 1 (LFA-1 or CD11a) promoter. Proc. Natl. Acad. Sci. USA.

[bib7] Davis D.M., Chiu I., Fassett M., Cohen G.B., Mandelboim O., Strominger J.L. (1999). The human natural killer cell immune synapse. Proc. Natl. Acad. Sci. USA.

[bib8] Grakoui A., Bromley S.K., Sumen C., Davis M.M., Shaw A.S., Allen P.M., Dustin M.L. (1999). The immunological synapse: a molecular machine controlling T cell activation. Science.

[bib9] Inlay M.A., Serwold T., Mosley A., Fathman J.W., Dimov I.K., Seita J., Weissman I.L. (2014). Identification of Multipotent Progenitors that Emerge Prior to Hematopoietic Stem Cells in Embryonic Development. Stem Cell Rev..

[bib10] Karlsson G., Rörby E., Pina C., Soneji S., Reckzeh K., Miharada K., Karlsson C., Guo Y., Fugazza C., Gupta R. (2013). The tetraspanin CD9 affords high-purity capture of all murine hematopoietic stem cells. Cell Reports.

[bib11] Kiel M.J., Yilmaz O.H., Iwashita T., Yilmaz O.H., Terhorst C., Morrison S.J. (2005). SLAM family receptors distinguish hematopoietic stem and progenitor cells and reveal endothelial niches for stem cells. Cell.

[bib12] Lu R., Neff N.F., Quake S.R., Weissman I.L. (2011). Tracking single hematopoietic stem cells in vivo using high-throughput sequencing in conjunction with viral genetic barcoding. Nat. Biotechnol..

[bib13] Morrison S.J., Wright D.E., Weissman I.L. (1997). Cyclophosphamide/granulocyte colony-stimulating factor induces hematopoietic stem cells to proliferate prior to mobilization. Proc. Natl. Acad. Sci. USA.

[bib14] Pruijt J.F., van Kooyk Y., Figdor C.G., Lindley I.J., Willemze R., Fibbe W.E. (1998). Anti-LFA-1 blocking antibodies prevent mobilization of hematopoietic progenitor cells induced by interleukin-8. Blood.

[bib15] Rossi D.J., Bryder D., Zahn J.M., Ahlenius H., Sonu R., Wagers A.J., Weissman I.L. (2005). Cell intrinsic alterations underlie hematopoietic stem cell aging. Proc. Natl. Acad. Sci. USA.

[bib16] Seita J., Weissman I.L. (2010). Hematopoietic stem cell: self-renewal versus differentiation. Wiley Interdiscip. Rev. Syst. Biol. Med..

[bib17] Spangrude G.J., Heimfeld S., Weissman I.L. (1988). Purification and characterization of mouse hematopoietic stem cells. Science.

[bib18] Van Epps D.E., Potter J., Vachula M., Smith C.W., Anderson D.C. (1989). Suppression of human lymphocyte chemotaxis and transendothelial migration by anti-LFA-1 antibody. J. Immunol..

[bib19] Wagers A.J., Allsopp R.C., Weissman I.L. (2002). Changes in integrin expression are associated with altered homing properties of Lin(-/lo)Thy1.1(lo)Sca-1(+)c-kit(+) hematopoietic stem cells following mobilization by cyclophosphamide/granulocyte colony-stimulating factor. Exp. Hematol..

[bib20] Weitz-Schmidt G., Welzenbach K., Brinkmann V., Kamata T., Kallen J., Bruns C., Cottens S., Takada Y., Hommel U. (2001). Statins selectively inhibit leukocyte function antigen-1 by binding to a novel regulatory integrin site. Nat. Med..

[bib21] Wright D.E., Cheshier S.H., Wagers A.J., Randall T.D., Christensen J.L., Weissman I.L. (2001). Cyclophosphamide/granulocyte colony-stimulating factor causes selective mobilization of bone marrow hematopoietic stem cells into the blood after M phase of the cell cycle. Blood.

