# Supplementary material for: Upregulation of CD11A on Hematopoietic Stem Cells Denotes the Loss of Long-Term Reconstitution Potential
Source: Stem Cell Reports. 2014 Oct 16;3(5):707–15. doi: 10.1016/j.stemcr.2014.09.007 (PMC4235136; doi:10.1016/j.stemcr.2014.09.007)
Supplement: Document S1. Supplemental Experimental Procedures, Figures S1–S4, and Table S1 [file mmc1.pdf]

Stem Cell Reports, Volume *3*

Supplemental Information

Upregulation of CD11A on Hematopoietic Stem Cells Denotes the Loss of Long-Term Reconstitution Potential

John W. Fathman, Nathaniel B. Fernhoff, Jun Seita, Connie Chao, Vanessa Scarfone, Irving L. Weissman, and Matthew A. Inlay

Supplemental Figures and Legends

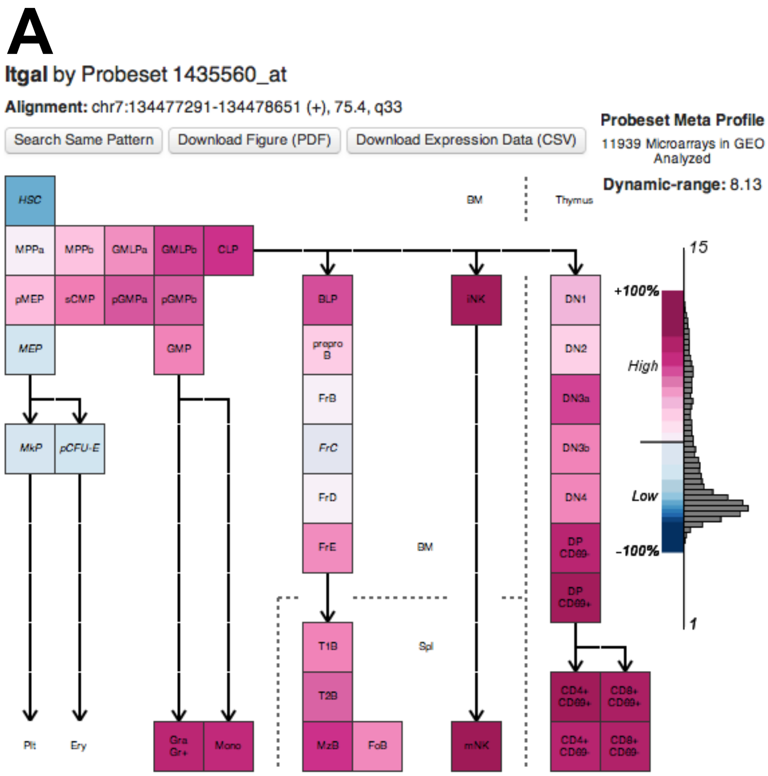

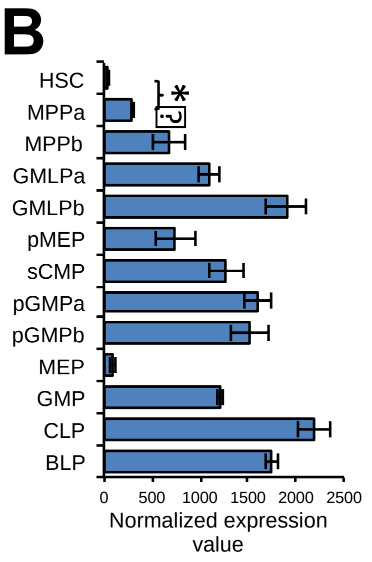

Figure S1. CD11A expression is downregulated in murine HSCs compared downstream progenitors (related to Figure 1). (**A**) Screenshot of a heat-map of CD11A expression across a panel of 39 hematopoietic populations (Seita et al., 2012). Normalized expression of a CD11A probeset (1435560_at) on Mouse 430 2.0 microarrays. Intensity of color indicates high (magenta) or low (blue) expression, as measured by percentile across a panel of nearly 11,939 microarrays of different mouse populations (right). Screenshot is taken from the Gene Expression Commons (gexc.stanford.edu). (**B**) Bar graph of CD11A microarray expression of HSCs and downstream progenitors. Normalized unlogged expression of the average CD11A expression among each population is shown. HSCs are from 4 microarrays, all others are from 3 arrays. Error bars are standard deviation. * indicates p value < 0.001 between HSC and MPPa (unpaired T-test). Please see supplemental experimental procedures for definitions of all populations. (**C**) Heat-map of cell cycle gene expression of 30 genes involved in cell cycle regulation (GO category "cell cycle") among microarrays of CD11A$^-$ and CD11A$^+$ HSC subfractions, as well as 11 downstream progenitor populations. Each row shows the relative expression of one cell cycle gene, with the intensity of colors corresponding to the percentile of expression.

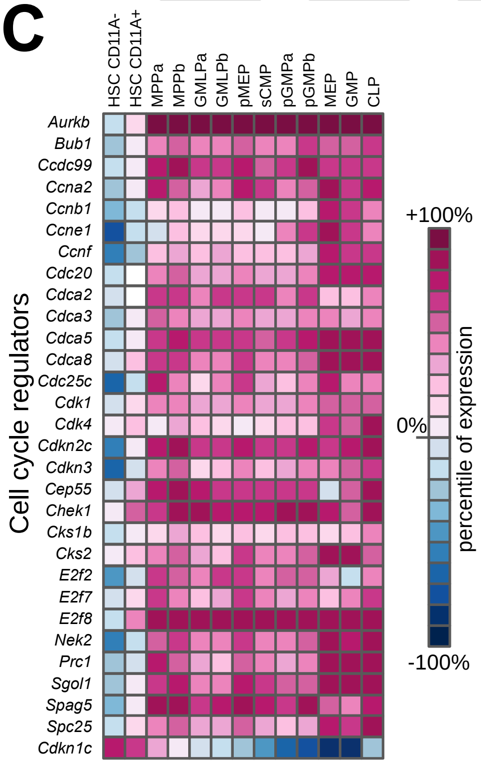

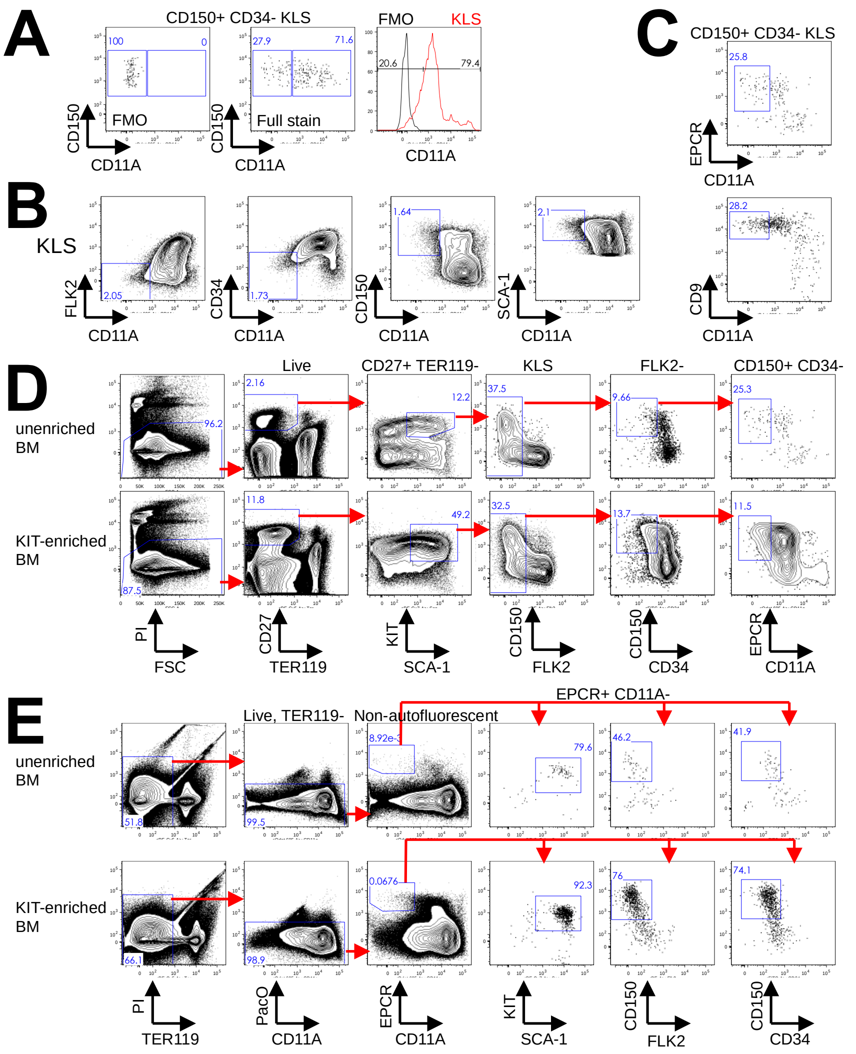

Figure S2. Guide to gating HSCs using CD11A expression (related to Figure 2). (**A**) FMO vs CD11A stain of BM HSCs. The histogram on the right shows the FMO (black) vs CD11A expression on KLS cells (red). (**B**) Comparison of CD11A to other HSC markers. CD11A (x-axis) versus other HSC markers (y-axis) of KLS cells is shown. (**C**) CD11A versus EPCR and CD9 on HSCs ($CD150^{+}$ $CD34^{-}$ KLS). Please note that the cutoff for $CD11a^{-}$ and $CD11A^{+}$ fractions is identical for all plots from S2A, S2B, and S2C. (**D**) CD11A/ EPCR expression on HSCs from unenriched (top row) versus KIT-enriched BM. The full gating strategy is shown. (**E**) EPCR and CD11A alone highly enrich for HSCs. Unenriched (top row) and KIT-enriched (bottom row) BM showing the purity of HSCs ($CD150^{+}$ $FLK2^{-}$ $CD34^{-}$ KLS) in the $EPCR^{+}$ $CD11A^{-}$ fraction. It is critical to gate out dead cells (PI^{+}), red blood cells ($TER119^{+}$) and autofluorescent cells ($PacO^{+}$), which have non-specific fluorescence in the EPCR/CD11A plot. In this case, the Pacific Orange (PacO) channel was empty and serves as a way to detect autofluorescent cells. Please note that the plots in S2D and S2E are from the same stain and only the order of gates is changed.

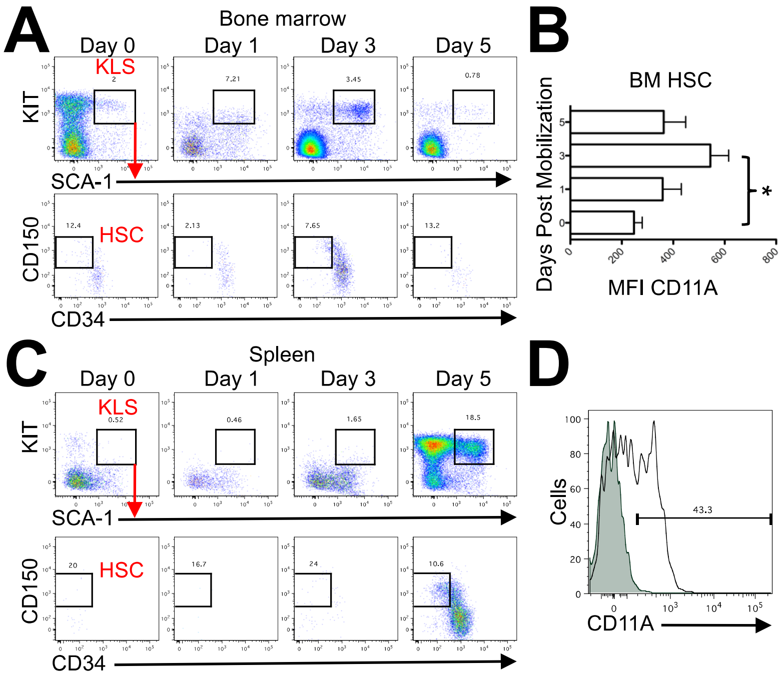

Figure S3. CD11A is upregulated on mobilized HSCs in the BM and spleen (related to Figure 3). Mobilized HSCs use these adhesion molecules to traffic from the bone marrow to the blood and subsequently the spleen. In G-CSF mobilization protocols, mice are initially injected intra-peritoneally with cyclophosphamide, which integrates into DNA and causes dividing cells to apoptose. Then on successive days sub-cutaneous injections of human G-CSF are administered. (**A**) Bone marrow of G-CSF mobilized mice were analyzed at indicated time points for expression of CD11A on HSCs. The plots in the top row were gated on Lin^- cells, and the bottow row on $Lin^-KIT^+SCA\text{-}1^+$ (KLS) cells. Gates and percentages of KLS (top row) and HSCs (bottom row) are shown. Typically, by day 1 after cyclophosphamide treatment, the majority of hematopoietic cells have died, except quiescent HSCs. By day 3, HSCs become activated by G-CSF, causing them to divide and differentiate in the bone marrow. (**B**) Mean fluorescence intensity (MFI) of CD11A expression on HSCs at different time points ($p<0.0008$). (**C**) Over the following days HSCs migrate out of the bone marrow and can be found in the blood and spleen. Spleens of G-CSF mobilized mice were analyzed at indicated time points for expression of CD11A on their HSCs. Gates are the same as in Fig. 4A. (**D**) Expression of CD11A on day 5 HSCs in the spleen of mobilized mice (grey: FMO, Black line: Day 5 spleen HSCs).

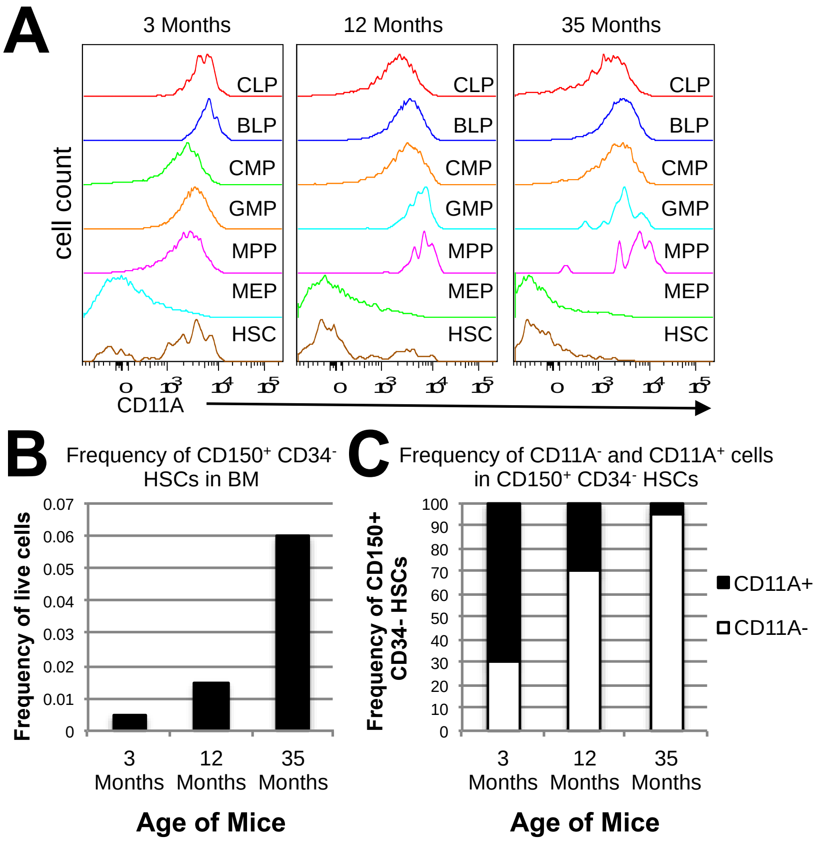

Figure S4. CD11A expression on aged HSCs (related to Figure 4). (**A**) CD11A expression profile of HSCs and downstream hematopoietic progenitors from young adult (3 month, left column), middle-aged (12 months, middle column), and old (35 months, right column) mice. (**B**) Frequency of HSCs in young, middle-aged, and old mice. (**C**) Frequency of CD11A$^-$ (white) and CD11A$^+$ (black) subfractions of HSCs in young, middle-aged, and old mice. Nearly all HSCs in old mice are CD11A$^-$.

Supplemental Experimental Procedures

Marker definitions of stem and progenitor populations used in microarray analyses

All populations used in microarray analyses are shown below and are as previously described (Seita et al., 2012). Lin = Lineage cocktail (CD3, CD4, CD8, B220, GR1, MAC1, TER119). CD11A$^-$ and CD11A$^+$ HSC populations were submitted to GEO in experiment GSE60597. Microarrays of all other populations have been previously published and submitted to GEO (GSE34723). Accession numbers for individual arrays are listed with each population in brackets.

CD11A$^-$ HSC (CD11A$^-$ Hematopoietic Stem Cell)
[GSM1483039, -40, -41]
CD27$^+$ Lin$^-$ KIT$^+$ SCA-1$^+$ FLK2$^-$ CD150$^+$ CD34$^-$ EPCR$^+$ CD11A$^-$

CD11A$^+$ HSC (CD11A$^+$ Hematopoietic Stem Cell)
[GSM1483042, -43]
CD27$^+$ Lin$^-$ KIT$^+$ SCA-1$^+$ FLK2$^-$ CD150$^+$ CD34$^-$ EPCR$^+$ CD11A$^+$

HSC (Hematopoietic stem cell, used in Figure S1A, S1B)
[GSM854035, -36, -37, -38]
CD34$^-$ FLK2$^-$ SCA-1$^+$ KIT$^+$ Lin$^-$

MPPa (Multi-potent Progenitor subset A)
[GSM854039, -40, -41]
CD34$^+$ CD150$^+$ TIE2$^+$ VCAM1$^+$ SCA-1$^+$ KIT$^+$ IL7Rα^- Lin$^-$

MPPb (Multi-potent Progenitor subset B)
[GSM854042, -43, -44]
CD34$^+$ CD150$^-$ TIE2$^+$ VCAM1$^+$ SCA-1$^+$ KIT$^+$ IL7Rα^- Lin$^-$

GMLPa (Granulo/Macrophage/Lymphoid Progenitor subset A)
[GSM854045, -46, -47]
CD34$^+$ CD150$^-$ TIE2$^-$ VCAM1$^+$ SCA-1$^+$ KIT$^+$ IL7Rα^- Lin$^-$

GMLPb (Granulo/Macrophage/Lymphoid Progenitor subset B)
[GSM854048, -49, -50]
CD34$^+$ CD150$^-$ TIE2$^-$ VCAM1$^-$ SCA-1$^+$ KIT$^+$ IL7Rα^- Lin$^-$

pMEP (pre-Megakaryocyte/Erythrocyte Progenitor)
[GSM854051, -52, -53]
CD34$^+$ FcγRII/III^{low} CD150$^+$ TIE2$^+$ VCAM1$^+$ SCA-1$^-$ KIT$^+$ IL7Rα^- Lin$^-$

sCMP (Strict Common Myeloid Progenitor)
[GSM854054, -55, -56]
CD34$^+$ FcγRII/III^{low} CD150$^-$ TIE2$^+$ VCAM1$^+$ SCA-1$^-$ KIT$^+$ IL7Rα^- Lin$^-$

pGMPa (pre-Granulocyte/Macrophage Progenitor subset A)
[GSM854057, -58, -59]
CD34$^+$ FcγRII/III^{low} CD150$^-$ TIE2$^-$ VCAM1$^+$ SCA-1$^-$ KIT$^+$ IL7Rα^- Lin$^-$

pGMPb (pre-Granulocyte/Macrophage Progenitor subset B)
[GSM854060, -61, -62]
CD34$^+$ FcγRII/III^{low} CD150$^-$ TIE2$^-$ VCAM1$^-$ SCA-1$^-$ KIT$^+$ IL7Rα^- Lin$^-$

MEP (Megakaryocyte/Erythrocyte Progenitor)
[GSM854063, -64, -65]
$CD34^-$ $Fc\gamma RII/III^-$ $SCA\text{-}1^-$ KIT^+ $IL7R\alpha^-$ Lin^-

GMP (Granulocyte/Macrophage Progenitor)
[GSM854066, -67, -68]
$CD34^+$ $Fc\gamma RII/III^+$ $SCA\text{-}1^-$ KIT^+ $IL7R\alpha^-$ Lin^-

CLP (Common Lymphoid Progenitor ($LY6D^-$))
[GSM507378, -79, -80]
Lin^- $CD11C^-$ $B220^-$ $CD27^+$ KIT^{int} $FLK2^+$ $IL7R\alpha^+$ $LY6D^-$

BLP (Earliest B-Lymphoid Progenitor)
[GSM854073, -74, -75]
Lin^- $CD11C^-$ $B220^-$ $CD27^+$ KIT^{int} $FLK2^+$ $IL7R\alpha^+$ $LY6D^+$

Microarray analysis
Microarrays (Affymetrix Mouse 430 2.0) of $CD11A^-$ and $CD11A^+$ subpopulations were generated as described (Seita et al., 2012). Microarrays were normalized to a panel of approximately 12,000 public microarrays, and the heat-maps indicate percentile of expression compared to the 12,000 arrays. Normalization and gene expression analyses were performed using the Gene Expression Commons (GEXC, gexc.stanford.edu). Microarray data included in this experiment can be accessed and analyzed on the GEXC under model 1007 (gexc.stanford.edu/model/1007).

Mobilization
Mice were injected intra-peritoneally with cyclo-phosphamide (200 mg/kg) (Sigma) and then on successive days with human G-CSF (Neupogen, 250 μg/kg) per day administered as a single daily sub-cutaneous injection. The day of cyclo-phosphamide treatment was considered day −1 and the first day of G-CSF treatment was counted as day 0. For example, mice sacrificed on day 3 of the mobilization protocol were sacrificed on the day after the third G-CSF injection

Liquid cultures
Single cells from $CD11A^-$ and $CD11A^+$ HSC subpopulations were sorted directly into individual wells of a 96-well round bottom plate containing DMEM/F12 media with 10% FBS. Growth factors SCF (Stem Cell Factor), Flt3L, IL-3, TPO (Thrombopoietin), and EPO (Erythropoietin) were added at 10 ng/ml each. Colonies were scored at day 12 of culture for number of colonies, size of colonies, and lineages produced.

Supplemental Table S1. Antibodies

Antigen	Clone	Conjugate	Source
B220	RA3-6B2	FITC	eBioscience
CD3	2C11	Alexa488	Weissman
	2C11	Alexa680	Weissman
	2C11	Pacific Blue	Weissman
	2C11	Pacific Orange	Weissman
CD4	GK1.5	Pacific Blue	Weissman
CD8	53.6.7	Pacific Blue	Weissman
CD9	MZ3	PE	Biolegend
CD11A (LFA-1)	M17/4	biotin	eBioscience
	M17/4	PE	Biolegend
CD11B (MAC1)	M1/70	PECy5	eBioscience
	M1/70	Pacific Blue	Weissman
	M1/70	Pacific Orange	Weissman
CD11C	N418	PECy5.5	eBioscience
	N418	APCCy7	eBioscience
CD16/32 (FcγRII/III)	93	APCCy7	Biolegend
CD19	1D3	PECy5.5	eBioscience
CD34	RAM34	FITC	eBioscience
CD45.1 (Ly5.1)	A20.1.7	Alexa680	Weissman
CD45.2 (Ly5.2)	AL1-4A2	PE	Weissman
CD117 (KIT)	2B8	APCCy7	eBioscience
CD127 (IL7Rα)	A7R34	PECy5	eBioscience
	A7R34	Pacific Blue	Weissman
	A7R34	biotin	eBioscience
CD135 (FLK2)	A2F10	PE	eBioscience
	A2F10	PECy5	eBioscience
	A2F10	biotin	eBioscience
CD150 (SLAMF1)	TC15-12F12.2	Alexa647	Biolegend
	TC15-12F12.2	PECy7	Biolegend
CD201 (EPCR)	eBio1560	PerCP-eFluor710	eBioscience
CD335 (NKp46)	29A1.4	FITC	eBioscience
	29A1.4	APC	eBioscience
GR1	8C5	Pacific Blue	Weissman
	8C5	Pacific Orange	Weissman
	A7R34	Pacific Blue	Weissman
	A7R34	biotin	eBioscience
Ki-67	16A8	PE	Biolegend
LY6D	49H4.3	Pacific Orange	Weissman
NK1.1	PK136	PECy5	eBioscience
	PK136	PECy7	eBioscience
SCA-1	D7	APC	eBioscience
	E13-161-7	Alexa680	Weissman
TER119	TER119	Pacific Orange	Weissman
	TER119	PECy5	eBioscience
Streptavidin		Qdot605	Invitrogen

“Weissman” denotes conjugations generated in the Weissman lab.

Supplemental References

Seita, J., Sahoo, D., Rossi, D.J., Bhattacharya, D., Serwold, T., Inlay, M.A., Ehrlich, L.I., Fathman, J.W., Dill, D.L., and Weissman, I.L. (2012). Gene Expression Commons: an open platform for absolute gene expression profiling. PLoS One *7*, e40321.
