# Supplementary material for: Upregulation of CD11A on Hematopoietic Stem Cells Denotes the Loss of Long-Term Reconstitution Potential
Source: Stem Cell Reports. 2014 Oct 16;3(5):707–15. doi: 10.1016/j.stemcr.2014.09.007 (PMC4235136; doi:10.1016/j.stemcr.2014.09.007)
Supplement: Document S2. Article plus Supplemental Information [file mmc2.pdf]

Stem Cell Reports
Report
ISSCR
OPEN ACCESS

Upregulation of CD11A on Hematopoietic Stem Cells Denotes the Loss of Long-Term Reconstitution Potential

John W. Fathman,[1,5,*] Nathaniel B. Fernhoff,[1] Jun Seita,[1] Connie Chao,[2] Vanessa M. Scarfone,[2] Irving L. Weissman,[1,3,4] and Matthew A. Inlay[1,2,4,*]
[1]Stanford Institute for Stem Cell Biology and Regenerative Medicine, Stanford University, Stanford, CA 94305, USA
[2]Sue and Bill Gross Stem Cell Research Center, Department of Molecular Biology and Biochemistry, University of California Irvine, Irvine, CA 92697, USA
[3]Ludwig Center for Cancer Stem Cell Research and Medicine, Stanford University, Stanford, CA 94305, USA
[4]Co-senior author
[5]Present address: Genomics Institute of the Novartis Research Foundation, San Diego, CA 92121, USA
*Correspondence: jfathman@gnf.org (J.W.F.), minlay@uci.edu (M.A.I.)
http://dx.doi.org/10.1016/j.stemcr.2014.09.007
This is an open access article under the CC BY license (http://creativecommons.org/licenses/by/3.0/).

SUMMARY

Small numbers of hematopoietic stem cells (HSCs) generate large numbers of mature effector cells through the successive amplification of transiently proliferating progenitor cells. HSCs and their downstream progenitors have been extensively characterized based on their cell-surface phenotype and functional activities during transplantation assays. These cells dynamically lose and acquire specific sets of surface markers during differentiation, leading to the identification of markers that allow for more refined separation of HSCs from early hematopoietic progenitors. Here, we describe a marker, CD11A, which allows for the enhanced purification of mouse HSCs. We show through in vivo transplantations that upregulation of CD11A on HSCs denotes the loss of their long-term reconstitution potential. Surprisingly, nearly half of phenotypic HSCs (defined as $Lin^-KIT^+SCA\text{-}1^+CD150^+CD34^-$) are $CD11A^+$ and lack long-term self-renewal potential. We propose that $CD11A^+Lin^-KIT^+SCA\text{-}1^+CD150^+CD34^-$ cells are multipotent progenitors and $CD11A^-Lin^-KIT^+SCA\text{-}1^+CD150^+CD34^-$ cells are true HSCs.

INTRODUCTION

Since their identification and isolation over 25 years ago (Spangrude et al., 1988), hematopoietic stem cells (HSCs) have arguably become the most well-characterized tissue-specific “adult” stem cell. HSCs reside atop the hematopoietic hierarchy and give rise to functional effector cells through a succession of increasingly committed downstream progenitor cell stages (Seita and Weissman, 2010). Our understanding of the molecular basis for lineage determination and self-renewal has depended critically on our ability to identify and isolate HSCs and their downstream progeny with high purity. HSCs are primarily quiescent, but their immediate downstream progeny, multipotent progenitors (MPPs), are transit-amplifying cells and rapidly proliferate and differentiate to replenish the blood supply. Thus, reliably separating HSCs from MPPs is key to characterizing their distinct self-renewal and differentiation potentials, and considerable attention has been paid to markers that can better separate these populations, which include SCA-1, KIT, CD34, and CD150 (Kiel et al., 2005). Analyses of purified HSCs transplanted into lethally irradiated mice at low numbers (1 to 50 cells per mouse) have revealed functional heterogeneity within phenotypic HSCs (Beerman et al., 2010; Benz et al., 2012; Lu et al., 2011). Beerman et al. demonstrated that higher levels of CD150 (SLAMF1) marked HSCs that are skewed toward myeloid cell fates, compared to $CD150^{int}$ HSCs, which display a more balanced lineage output (Beerman et al., 2010). Other groups have shown heterogeneity of HSCs using a variety of markers such as cytokine receptors, other Slam family members, and adhesion molecules (Arai et al., 2004; Kiel et al., 2005; Wagers et al., 2002). Thus, even with the existing panel of markers, the HSC population is likely heterogeneous.

Based on our own gene expression analyses of HSCs and downstream progenitors (Seita and Weissman, 2010), we identified integrin alpha L (CD11A, *Itgal*) as a possible marker to better purify HSCs. CD11A heterodimerizes with CD18 (integrin beta-2) to form the adhesion molecule LFA-1 (lymphocyte function-associated anigten-1) (Cornwell et al., 1993). LFA-1 is expressed on all leukocytes and plays important roles in many immunological processes, including transendothelial migration toward sites of inflammation (Van Epps et al., 1989), lymphocyte costimulation and effector-target cell interactions (Davis et al., 1999), and formation of the T cell immunological synapse (Grakoui et al., 1999).

In this study, we show that CD11A has bimodal expression on phenotypic HSCs ($Lin^-KIT^+SCA\text{-}1^+FLK2^-CD150^+CD34^-$). Our data show that the $CD11A^-$ fraction of HSCs contains all functional HSC activity, with the $CD11A^+$ fraction composed of more differentiated cells that lack long-term self-renewal activity.

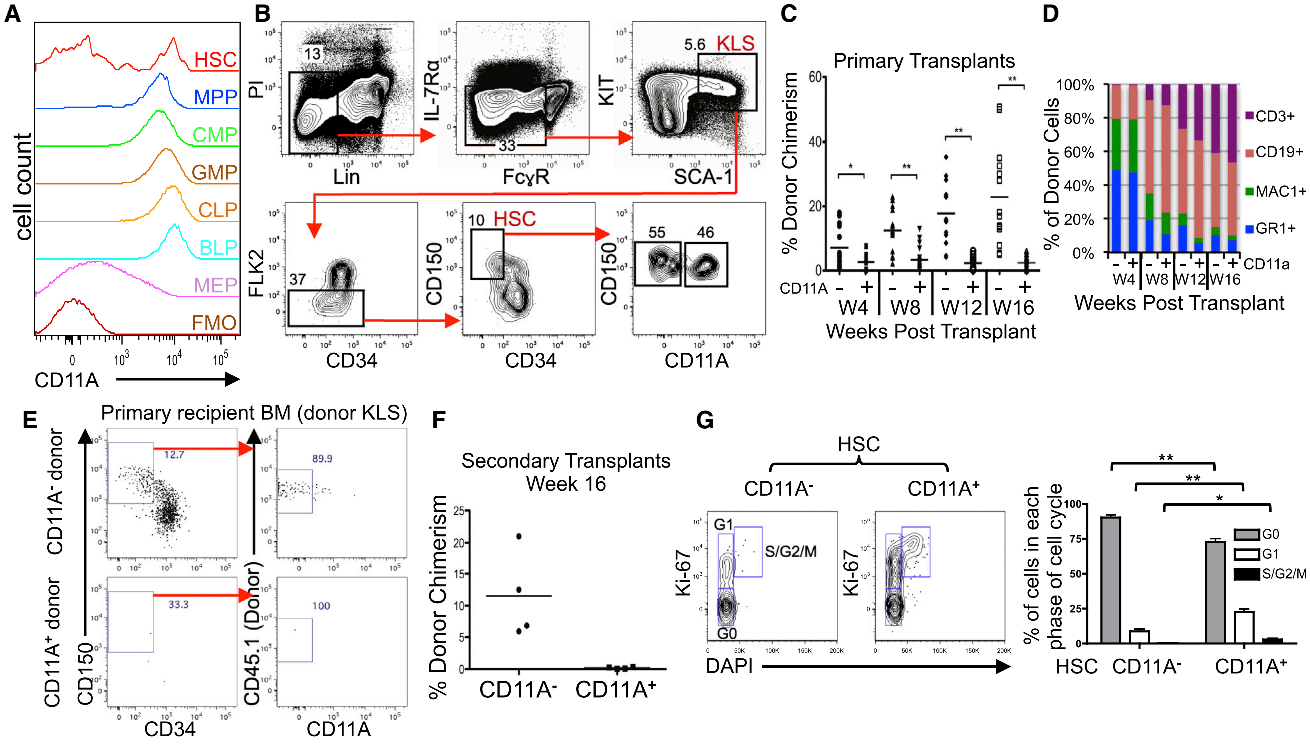

Figure 1. Bimodal Expression of CD11A on Phenotypic HSCs

(A) BM populations were analyzed for cell-surface expression of CD11A. Fluorescence minus one (FMO) was used as the negative control, gated on Lin^- cells.

(B) Gating scheme of murine HSCs. Phenotypic HSCs are gated on live cells (PI^-), Lin^- ($CD3\varepsilon^-$, $CD19^-$, $NK1.1^-$, $GR1^-$, $TER119^-$), $IL\text{-}7R\alpha^-$, $Fc\gamma R^{lo}$, KIT^+, $SCA\text{-}1^+$, $FLK2^-$, $CD150^+$, and $CD34^-$. The markers IL-7Rα and FcγR are typically not necessary to identify HSCs but are shown here for additional resolution.

(C) $CD11A^-$ (–) and $CD11A^+$ (+) HSC subfractions (50 cells/mouse) were transplanted into five lethally irradiated congenic recipients along with 2×10^5 competitive BM cells. Mice were analyzed at 4 (W4), 8 (W8), 12 (W12), and 16 (W16) weeks after transplantation for total donor blood chimerism (*$p < 0.01$, **$p < 0.0001$). Graph includes data from three independent experiments.

(D) Donor-derived lineage distribution for granulocytes ($MAC1^+GR1^+$, "GR1," blue), macrophages ($MAC1^+GR1^-$, "MAC1," green), B cells ($CD19^+$, red), and T cells ($CD3\varepsilon^+$, purple) from $CD11A^-$ (–) and $CD11A^+$ (+) HSC subfractions.

(E) BM from primary recipient mice transplanted with the $CD11A^-$ and $CD11A^+$ HSC subfractions were analyzed for CD11A expression in donor-derived HSC. Only donor-derived KLS cells ($Lin^-KIT^+SCA\text{-}1^+$) are shown.

(F) BM from primary recipients transplanted with $CD11A^-$ and $CD11A^+$ HSC subfractions were harvested at 16 weeks posttransplant, and 2×10^5 cells were retransplanted into lethally irradiated congenic secondary recipients. Blood was analyzed 16 weeks posttransplant for long-term engraftment.

(G) Cell-cycle analysis of $CD11A^-$ and $CD11A^+$ HSC subfractions. BM was stained with Ki-67 and DAPI to identify the percentage of $CD11A^-$ and $CD11A^+$ HSCs in G0 ($Ki\text{-}67^-$ $DAPI^-$), G1 ($Ki\text{-}67^+$ $DAPI^-$) and S/G2/M ($Ki\text{-}67^+$ $DAPI^+$) phases. Statistics are Student's unpaired t test ($n = 4$, *$p < 0.05$, **$p < 0.01$).

RESULTS AND DISCUSSION

Bimodal Expression of CD11A on Phenotypic HSCs in Mice

Based on a screen of a microarray database spanning over 35 mouse hematopoietic populations (Seita and Weissman, 2010), we discovered that HSCs express much lower levels of CD11A than downstream progenitors (Figures S1A and S1B available online). We examined mouse whole bone marrow (BM) with anti-CD11A antibodies (Abs) to measure CD11A surface expression by flow cytometry (Figure 1A). All mature lymphocytes were positive for CD11A on their cell surface (data not shown), and almost all hematopoietic progenitor populations expressed high levels of CD11A, including MPPs and both myeloid (CMP, GMP) and lymphoid (CLP, BLP) committed progenitors (Figure 1A, see Supplemental Experimental Procedures for definitions and surface marker phenotypes). Only the megakaryocyte/erythrocyte progenitor (MEP) expressed low levels of CD11A.

In contrast, HSCs (defined as Lin$^-$KIT$^+$SCA-1$^+$FLK2$^-$ CD150$^+$CD34$^-$) had a bimodal expression of CD11A (Figures 1A and 1B). The CD11A$^-$ fraction accounts for anywhere from 30%–70% of the phenotypic HSC population, depending on the strain and age of the mouse (Figure 1B).

CD11A$^-$ Fraction Enriches for HSC Activity

The gold standard assay to functionally identify HSCs is long-term multilineage reconstitution after intravenous transplantation into lethally irradiated mice. We transplanted 50 cells from the phenotypic HSC CD11A$^-$ and CD11A$^+$ subpopulations into lethally irradiated congenic recipients and analyzed the blood for total donor chimerism and lineage distribution every 4 weeks (Figures 1C and 1D). At all time points, the CD11A$^-$ fraction gave a higher burst size compared to CD11A$^+$ fraction (Figure 1C). This trend magnified over the 16 week time course, with the contribution of the CD11A$^-$ fraction increasing over time. Conversely, the median chimerism from the CD11A$^+$ fraction decreased overall during this span. We also examined the lineage distribution of donor cells in the recipient blood and observed no difference in lineage bias between the CD11A$^-$ and CD11A$^+$ subfractions of HSCs (Figure 1D).

Based on the phenotypic similarities between the two subpopulations and the similarity in lineage potentials, we hypothesized that the CD11A$^-$ fraction is upstream of, and gives rise to, the CD11A$^+$ fraction. In support of this notion, the CD11A$^-$ HSC subfraction could give rise to both CD11A$^-$ and CD11A$^+$ HSC subtypes in the BM of primary recipients, whereas we saw no phenotypic HSCs in the donor BM population from the CD11A$^+$ fraction transplants (Figure 1E).

The most definitive test of self-renewal capability is demonstrated in secondary transplants. We therefore isolated BM from the primary recipients of CD11A$^-$ and CD11A$^+$ transplants and retransplanted them into secondary hosts (Figure 1F). When we analyzed the secondary hosts 16 weeks after transplant, we only detected donor-derived cells from the CD11A$^-$ HSC fraction, proving they have functional self-renewal activity.

HSCs, which are mainly quiescent, likely have significant differences in the expression of cell-cycle regulators compared to the more robustly proliferative MPPs. We compared gene expression microarrays of these HSC subpopulations to existing arrays of MPPs and downstream progenitors for expression of cell-cycle genes (Figure S1C). Our data indicate that among all populations, the CD11A$^-$ HSC subfraction had the lowest expression of key cell-cycle regulators, including cyclins and cyclin-dependent kinases. The CD11A$^+$ fraction appeared to upregulate many of these cell-cycle promoters, expressing levels between that of CD11A$^-$ HSCs and downstream multipotent progenitors. In addition, we examined the cell-cycle status of CD11A$^-$ and CD11A$^+$ fractions of HSCs and determined that the CD11A$^-$ fraction had significantly greater frequency of cells in G0, and significantly fewer in G1 and S/G2/M phase (Figure 1G).

When examining FMO (fluorescence minus one) controls, it did not appear that CD11A was completely unexpressed in the CD11A$^-$ fraction of HSCs (Figure S2A), making it difficult to gate these HSC subfractions based on the FMO. However, by comparing CD11A expression to other HSC markers, such as CD34, FLK2, and CD150, a clear population can be identified (Figure S2B). We also compared CD11A expression on HSCs to two new HSC markers EPCR (*Procr*, CD201) and CD9 (Balazs et al., 2006; Karlsson et al., 2013) and found that CD11A was able to identify a population that would not be separable using either EPCR or CD9 (Figure S2C). However, we did find that EPCR in combination with CD11A could clearly identify a subset of EPCR$^+$ CD11A$^-$ HSCs (Figure S2D). In fact, EPCR and CD11A alone could highly enrich for HSCs from whole BM, at approximately 40% purity using all other HSC markers (Figure S2E). If BM is first enriched for KIT$^+$ cells using anti-KIT microbeads, the purity of HSCs improves to 74% within the EPCR$^+$ CD11A$^-$ fraction (Figure S2E).

All HSCs Are within the CD11A$^-$ Fraction of BM

It is possible that, because of the high number of markers and stringent sorting criteria we used to purify HSCs, we may have missed functional HSCs that fall outside of our gates that may be CD11A$^+$. To determine whether any long-term reconstituting activity exists within CD11A$^+$ BM cells, we sorted whole BM based only on CD11A expression into positive and negative fractions and transplanted the entirety of each fraction into recipient mice (Figures 2A–2C). We designed our transplants to be competitive, sorting CD11A$^-$ cells from GFP$^+$ BM and CD11A$^+$ cells from CFP$^+$ BM (and vice versa) and then cotransplanting them into the same recipients (Figure 2B). At 4, 8, 12, and 24 weeks posttransplant, we identified donor cells from both CD11A$^-$ and CD11A$^+$ fractions in the recipient peripheral blood (Figure 2C). However, donor granulocytes were only from the CD11A$^-$ fraction. Because granulocytes are short lived, they are a better indicator of HSC engraftment than longer-lived cells such as lymphocytes. Furthermore, when we examined the BM of recipient animals at 24-weeks, only CD11A$^-$ BM gave rise to donor HSCs (Figure 2C). Our data clearly indicate that all HSCs reside within the CD11A$^-$ fraction of BM.

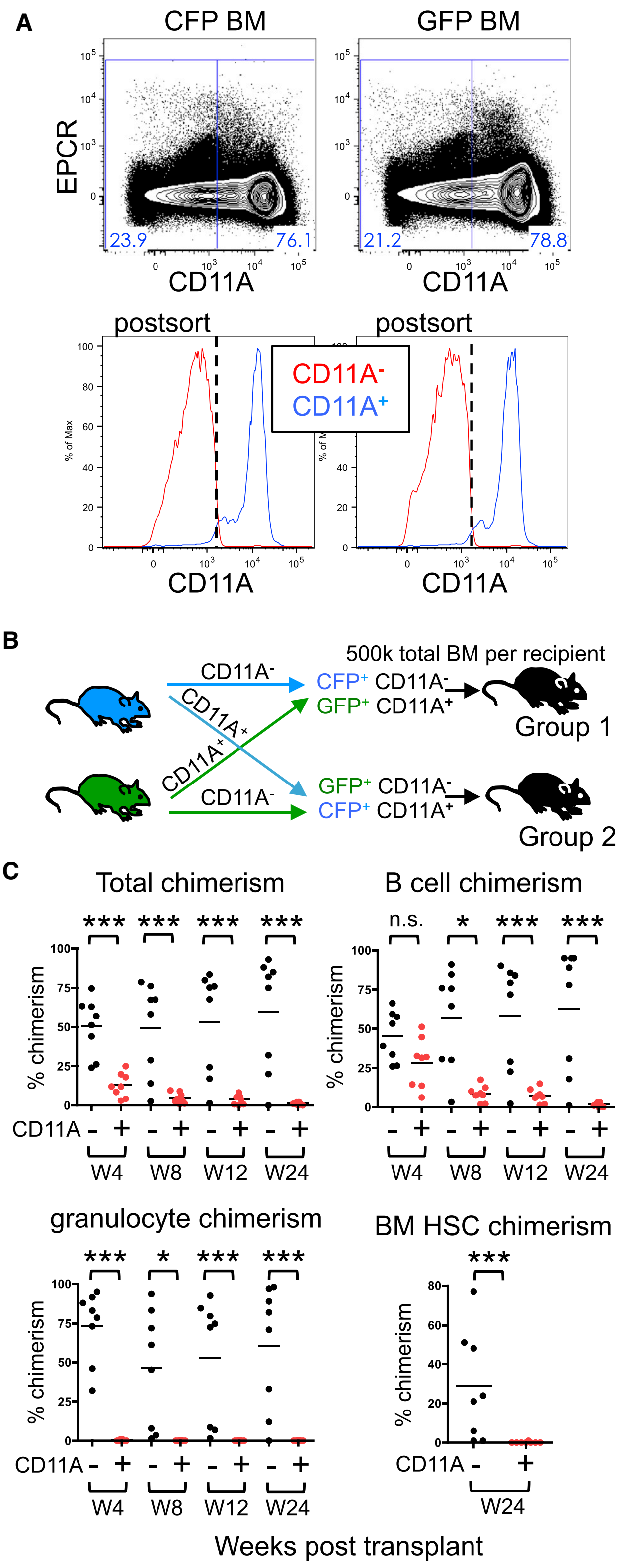

Figure 2. CD11A Antibody Separates BM Reconstituting Activity

(A) CFP^+ and GFP^+ BM cells were sorted based on CD11A expression into $CD11A^-$ (red) and $CD11A^+$ (blue) fractions.

(B) Recipient mice were transplanted with $CD11A^-$ and $CD11A^+$ fractions in two groups. Group 1 received CFP^+ $CD11A^-$ and GFP^+ $CD11A^+$ cells. Group 2 received GFP^+ $CD11A^-$ and CFP^+ $CD11A^+$ cells. All mice received a total of 500,000 cells at the physiologic ratio of $CD11A^-$ and $CD11A^+$ BM cells.

(C) Time-course analysis of donor chimerism. Donor chimerism derived from $CD11A^-$ and $CD11A^+$ BM cells is indicated for total cells (upper left), B cells (upper right), and granulocytes (lower left) in the peripheral blood at 4, 8, 12, and 24 weeks posttransplant, and HSCs (lower right) in the BM at 24 weeks. Data from groups 1 and 2 are pooled, and chimerism from $CD11A^-$ fraction (–, black circles) is shown on the left, and $CD11A^+$ fraction (+, red circles) is shown on the right at each time point. Percentages are out of total cells, including those of the host. *$p < 0.05$, ***$p < 0.001$ (Student's unpaired t test).

Anti-CD11A Antibody Does Not Inhibit Engraftment of HSCs or Homing of $CD11A^+$ Cells into the BM

LFA-1 (CD11A/CD18) binds to ICAMs on the vascular lumen to help mediate extravasation (Van Epps et al., 1989). HSCs injected intravenously must migrate from the blood into the BM to engraft. It is known that the CD11A Ab clone we used can block LFA-1 binding to ICAMs (Weitz-Schmidt et al., 2001). Therefore, we next determined if blocking CD11A function with the CD11A Ab inhibited migration into the bone marrow and caused the low engraftment rates in the $CD11A^+$ fraction we observed by transplantation. We first tested whether the CD11A Ab could block short-term homing of $CD11A^+$ cells to the BM (Figures 3A–3D). We determined that 100 ng of CD11A Ab was sufficient to saturate 10^6 BM cells (Figure 3A). We then harvested BM from CFP^+ mice and stained them with CD11A Ab (with 100 ng/10^6 cells), or left them untreated. We intravenously transplanted 10 million cells per recipient into unirradiated GFP^+ mice and then analyzed the BM 3 hr posttransplant for donor (CFP^+) cells. We found no significant difference in the percent of CFP^+ donor cells in the bones between untreated and CD11A Ab-treated BM (Figures 3B and 3C). The distribution of donor lineages that homed to the BM was also equivalent, with the majority of homed cells being granulocytes and B cells, both of which express high levels of CD11A (Figure 3D). Thus, the CD11A Ab does not appear to block immediate homing to the BM of $CD11A^+$ cells.

We next tested whether long-term engraftment was inhibited by CD11A Ab. We purified HSCs without the use of the CD11A Ab and then split the purified HSCs into two groups: one was treated with anti-CD11A Ab and the other with an isotype control (Figure 3E). We then transplanted 100 HSCs from each group and analyzed blood every 4 weeks for donor chimerism. At no point did we observe a statistical difference in donor chimerism between the two groups, demonstrating that the CD11A Ab did not

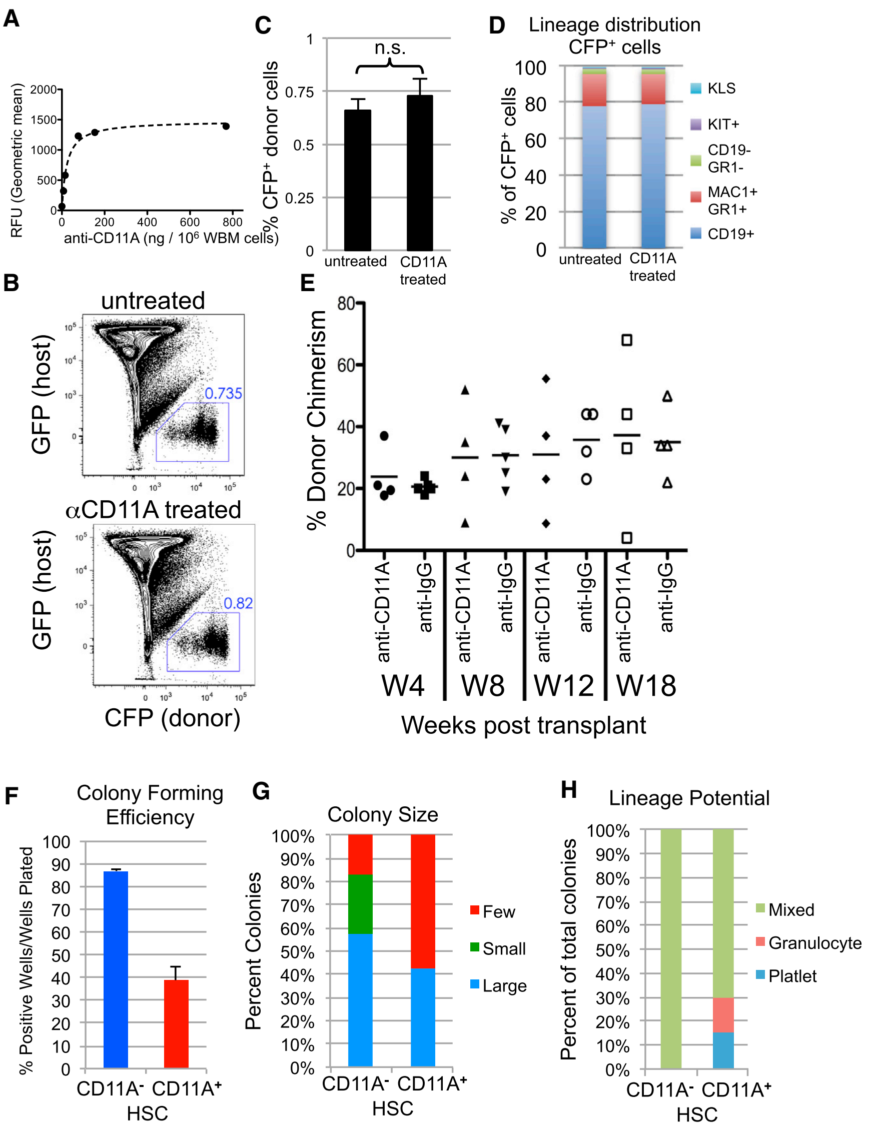

Figure 3. CD11A Antibody Does Not Inhibit BM Homing or Long-Term Engraftment

(A) Saturation curve for anti-CD11A Ab on BM cells.

(B and C) CFP$^+$ bone marrow cells (10×10^6/mouse) were untreated or stained with CD11A Ab and then transplanted into GFP$^+$ unirradiated recipients (n = 5 each group). Three hours after transplantation, bones were harvested and analyzed for homing of CFP$^+$ donor cells. (B) Representative BM from GFP hosts transplanted with untreated (top) and CD11A-Ab-treated (bottom) CFP$^+$ BM. Live cells are shown. (C) Comparison of the percentage of CFP$^+$ donor cells from untreated or CD11A-Ab-treated BM. Error bars are SD (n = 5) and are not significant (n.s.).

(D) Lineage distribution of CFP$^+$ donor-BM from untreated (left) and CD11A-Ab-treated (right) BM. The percentage of each lineage among donor-derived cells is shown. "KLS" is KIT$^+$Lin$^-$SCA-1$^+$.

(E) Phenotypic HSCs (Lin$^-$KIT$^+$SCA-1$^+$FLK2$^-$ CD150$^+$CD34$^-$) were purified and split into two groups, one was treated with anti-CD11A, and the other was treated with Rat anti-IgG isotype control. One hundred HSCs from each group were transplanted into four lethally irradiated congenic mice along with 2×10^5 competitive BM cells. Mice were analyzed every 4 weeks for blood donor chimerism levels up to 4 months. Bars indicate average percentage of donor chimerism. Data are representative of two experiments.

(F) Single-cell liquid cultures of CD11A$^-$ (blue, left) and CD11A$^+$ (red, right) HSC subfractions. The percentage of wells with colonies at day 12 is shown. Error bars are SD (n = 3 experiments).

(G) The size of colonies at day 12 (large >1,000 cells, small <1,000 cells, few less than ten cells).

(H) Distribution of the lineages produced by CD11A$^-$ and CD11A$^+$ subpopulations in vitro. For in vitro assays, each subpopulation was clone sorted into one 96-well plate. Data are representative of three independent experiments.

inhibit engraftment, and that the inability of the CD11A$^+$ fraction to engraft was not due to blocking LFA-1/ICAM interactions (Figure 3E).

To test the robustness of CD11A$^-$ and CD11A$^+$ HSC fractions' regenerative capacity in the absence of transplantation, we used in vitro culture methods to assess the colony formation efficiency of the two populations, thus bypassing any homing or engraftment issues. Single cells were sorted directly into wells with media and cytokines (SCF, Flt3L, interleukin-3 [IL-3], TPO, EPO) and cultured for up to 12 days. The CD11A$^-$ subpopulation displayed greater colony size (Figure 3F), colony forming efficiency (Figure 3G), and lineage potential (Figure 3H) than the CD11A$^+$ subpopulation.

HSCs Upregulate CD11A Expression during Granulocyte Colony-Stimulating Factor Mobilization

Administration of growth factors can activate HSCs and cause them to mobilize into the blood stream. Previous studies in mice have shown that mobilized HSCs in the periphery have elevated levels of several adhesion molecules (Wagers et al., 2002), and that pretreatment with anti-LFA-1 Abs inhibited the migration of HSCs out of the BM following IL-8-induced mobilization (Pruijt et al., 1998).

We hypothesized that CD11A would be an important integrin in HSC mobilization and examined CD11A expression on BM and splenic HSCs following granulocyte colony-stimulating factor (G-CSF) mobilization (Figure S3). Indeed, CD11A increased as HSCs proliferated in the BM, peaking at day 3, just prior to HSC migration into the blood (Figure S3B). Moreover, HSCs in the spleen at day 5 showed similarly high CD11A expression as the BM HSCs did on day 3 (Figure S3D) (Morrison et al., 1997; Wright et al., 2001). Compared to the steady-state BM, where CD11A expression appears to mark the loss of self-renewal potential of HSCs, in the context of mobilization, it seems that HSCs upregulate CD11A in order to exit from the niche but still retain their reconstitution capacity.

Frequency of the CD11A$^-$ Subfraction of HSCs Increases with Age

Although HSC function declines with age, the frequency of phenotypic HSCs increases (Rossi et al., 2005). We next compared CD11A expression in middle-aged (12 months) and old mice (35 months), to young adult mice (3 months) (Figure S4). Although all progenitor populations except MEP appeared to uniformly express CD11A regardless of age, we found that within the HSC population, the fraction of CD11A$^-$ cells increased with age (Figures S4A and S4C). At 35 months, almost all HSCs (>95%) are CD11A$^-$. This correlates with the increase in phenotypic HSCs observed in aged mice (Figure S4B).

Both CD11A$^-$ and CD11A$^+$ HSC Subpopulations from e17.5 Fetuses Can Engraft Long Term

If the frequency of the CD11A$^-$ fraction increased with age, we hypothesized that younger mice would have a decreased fraction of CD11A$^-$ HSCs. We examined HSCs from 3-day-old neonates, as well as from embryonic day 17.5 (e17.5) fetal liver (FL), which is a site of embryonic hematopoiesis, and found that the fraction of CD11A$^+$ phenotypic HSC was greater in e17.5 FL than in neonates or adult mice (Figure 4A). We next sorted and transplanted CD11A$^-$ and CD11A$^+$ subfractions of phenotypic HSCs from e17.5 FL (Figure 4B). Because the engraftability of embryonic HSCs is less than that from adults, we transplanted 450 cells of each. Surprisingly, we observed long-term donor chimerism from both CD11A$^-$ and CD11A$^+$ HSC subfractions (Figure 4B), with no differences in lineage distribution (Figure 4C). When we examined the recipients' BM 14 weeks after transplantation, we found that both CD11A$^-$ and CD11A$^+$ donor e17.5 HSCs could give rise to both CD11A$^-$ and CD11A$^+$ HSCs in the recipients' BM (Figure 4D). This indicates that at e17.5, functional HSCs are found within both CD11A$^-$ and CD11A$^+$ fractions. However, we then resorted donor-derived CD11A$^-$ and CD11A$^+$ phenotypic HSCs from the primary recipients for secondary transplants and found that within the secondary recipients, only the CD11A$^-$ fraction engrafted, regardless of whether they originally came from CD11A$^-$ or CD11A$^+$ e17.5 HSCs (Figure 4E). Our data suggest that during embryonic development, long-term engraftable HSCs can come from either the CD11A$^-$ or CD11A$^+$ fraction, but, once engrafted in the adult bone marrow, only the CD11A$^-$ fraction retains engraftability. In a related study, we show that all clonal multilineage potential in the embryo from e9.5 to e11.5 is contained within a similar CD11A$^-$ population (Inlay et al., 2014). However, at e12.5 we detected multilineage potential from both CD11A$^-$ and CD11A$^+$ cells, consistent with our results here that engraftable HSCs are present in both the CD11A$^-$ and CD11A$^+$ fractions of HSCs in the embryo. In the embryo, hematopoiesis transitions through multiple sites including the yolk sac, dorsal aorta, fetal liver, and bone marrow (Christensen et al., 2004), and we hypothesize that CD11A may play an important role in the migration of embryonic HSCs and/or their precursors through each site.

In this study, we have discovered that only half of mouse phenotypic HSCs, as defined by the most stringent criteria (Lin$^-$KIT$^+$SCA-1$^+$FLK2$^-$CD150$^+$CD34$^-$), express CD11A, and that only the CD11A$^-$ fraction of HSCs possesses the property of long-term multilineage differentiation and self-renewal upon intravenous transplantation into adult mice. As such, our data indicate that nearly half of the cells currently isolated as HSCs are not functional HSCs. This finding has clear implications for how we molecularly characterize HSCs and whether we can identify with certainty HSC niches in mouse bone marrow.

EXPERIMENTAL PROCEDURES

Animals

All animal procedures were approved by the International Animal Care and Use Committee (IACUC) and the Stanford Administrative Panel on Laboratory Animal Care (APLAC). We used C57Bl/Ka-THY1.2 CD45.2 (B/Ka), C57Bl/Ka-THY1.1 CD45.2 (BA), and C57Bl/6-THY1.2 CD45.1 (CD45.1). Strains were derived and maintained in the I.L.W.'s laboratory.

Antibodies

All antibodies, including clones and conjugations, used in this study are listed in Table S1.

Cell Sorting

Bone marrow was harvested from donor mice by crushing bones and removing debris on a density gradient using Histopaque 1077 (Sigma). Where indicated, bone marrow was KIT enriched

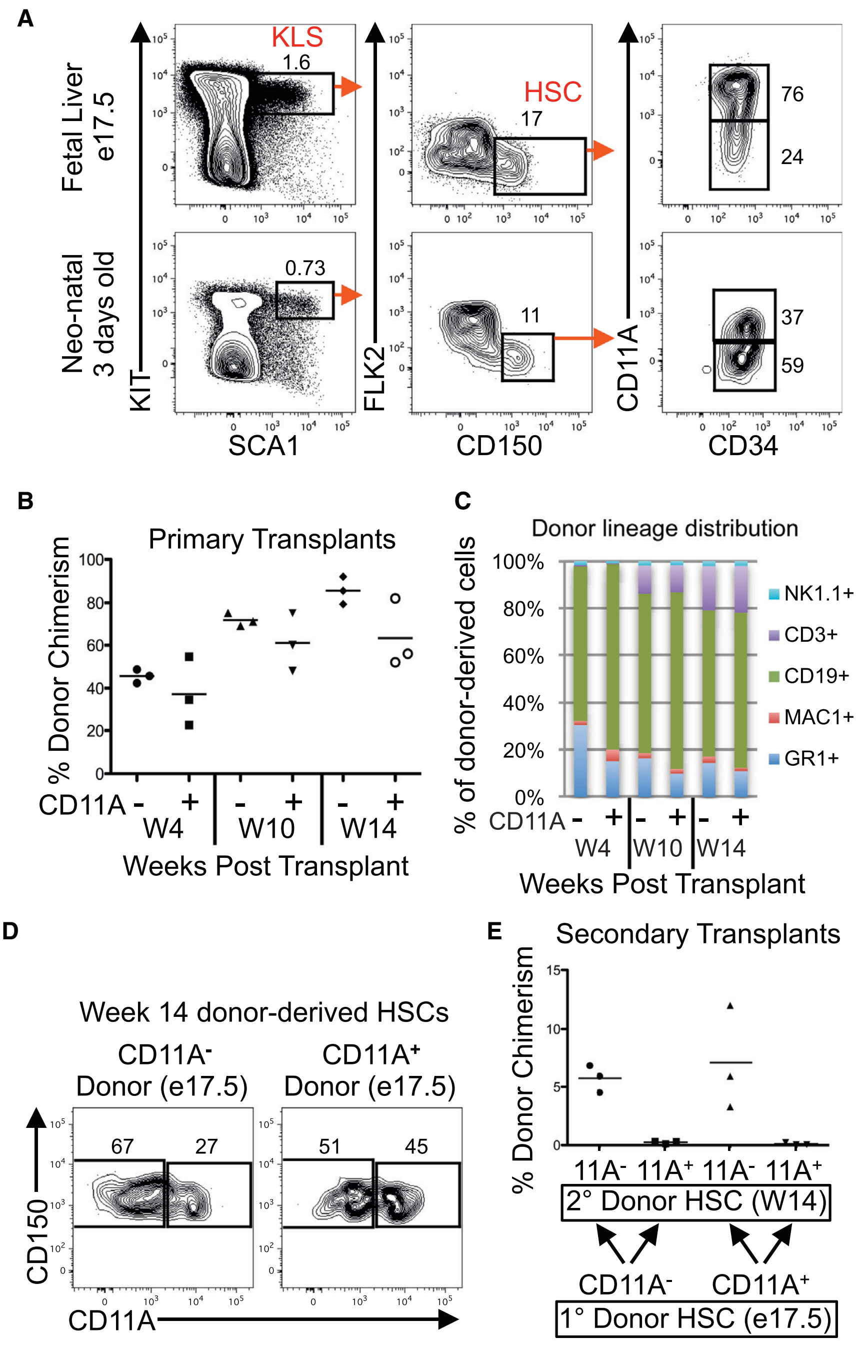

Figure 4. CD11A Expression on Fetal Liver and Neonate HSCs

(A) HSC stains of e17.5 fetal liver (top row) and 3-day-old neonates (bottom row). Gates and percentages of KLS, HSC, and $CD11A^{-}$ and $CD11A^{+}$ subpopulations are shown.

(B) Percentage of donor chimerism of $CD11A^{-}$ and $CD11A^{+}$ HSC subfractions sorted from e17.5 FL 4, 10, and 14 weeks after transplant. Bars indicate mean chimerism (n = 3).

(C) Lineage distribution of donor-derived cells 4, 10, and 14 weeks after transplantation.

(D) $CD11A^{-}$ and $CD11A^{+}$ donor-derived HSC subfractions from recipient mice 14 weeks after transplantation of $CD11A^{-}$ (left) and $CD11A^{+}$ (right) HSC subfractions from e17.5 FL.

(E) Percentage of donor chimerism from secondary transplants of $CD11A^{-}$ and $CD11A^{+}$ donor-derived HSC subfractions reharvested from week 14 recipient mice. The two left columns are from the $CD11A^{-}$ HSC subpopulation obtained from e17.5 FL, and the two right columns are from the $CD11A^{+}$ HSC subpopulation obtained from e17.5 FL.

using anti-KIT (CD117) microbeads on an AutoMACS (Miltenyi Biotec). Cells were stained with Abs listed in Table S1 in PBS with 2% FCS. All cells were double sorted on a BD FACS-Aria II (Becton Dickinson). FlowJo software (Tree Star) was used for data analysis. Cells were sorted into ice-cold PBS with 2% FCS, or into tissue culture medium, or TRIzol (Invitrogen). For the cell-cycle analysis, KIT-enriched BM was stained with Abs for surface markers, fixed in 4% paraformaldehyde, and permeabilized with BioLegend Perm/Wash Buffer according to the manufacturer's instructions. Cells were then stained with anti-Ki-67 (BioLegend), washed, and incubated with 1 μM DAPI for 10 min prior to analysis on a BD FACS Aria-II.

Transplantation

Fifty to 450 HSCs (as indicated) were transplanted by retro-orbital injection into isofluorane-anesthetized recipients that had been lethally irradiated (900 rads, single dose) alongside 2×10^5 helper bone marrow from congenically distinguishable wild-type mice. Blood was obtained from the tail vein of transplanted mice at

various time points, and erythrocytes were sedimented using 2% dextran in PBS at 37°C for 30 min. Cells were stained with lineage Abs and analyzed on the BD FACSAria-II.

ACCESSION NUMBERS

The GEO accession numbers for the microarrays of the $CD11A^{-}$ HSC population are GSM1483039, GSM1483040, and GSM148041 and for the $CD11A^{+}$ HSC population are GSM1483042 and GSM1483043 and are contained in experiment GSE34723.

SUPPLEMENTAL INFORMATION

Supplemental Information includes Supplemental Experimental Procedures, four figures, and one table and can be found with this article online at http://dx.doi.org/10.1016/j.stemcr.2014.09.007.

AUTHOR CONTRIBUTIONS

Experiments were designed by J.W.F. and M.A.I., advised by I.L.W., and performed by J.W.F., M.A.I., N.B.F., C.C., and V.M.S. J.S. contributed bioinformatics support and unpublished data. J.W.F. and M.A.I. wrote the manuscript, and I.L.W. edited it. Cell-cycle analysis was performed in the laboratory of M.A.I. All other experiments were performed in the laboratory of I.L.W.

ACKNOWLEDGMENTS

The authors wish to thank Agnieszka Czechowicz, Deepta Bhattacharya, Tom Serwold, and Debashis Sahoo for technical assistance and helpful discussion, and Elizabeth Zuo and the Gene Expression Core of the Stanford Protein and Nucleic Acid (PAN) facility for microarray processing. This study was supported by NIH grants R01HL058770, R01CA86085, and U01HL099999 (to I.L.W.), F31AG032854 (to J.W.F), and T32AI07290 (to J.W.F., M.A.I., and N.B.F.) and California Institute for Regenerative Medicine grants RT2-02060 (to I.L.W.), T1-00001 (to M.A.I. and J.S.), and CL1-00520-1.2 (to V.M.S. and the UC Irvine Stem Cell FACS Core), and the Virginia and D.K. Ludwig Fund for Cancer Research (to I.L.W).

Received: July 30, 2013
Revised: September 8, 2014
Accepted: September 9, 2014
Published: October 16, 2014

REFERENCES

Arai, F., Hirao, A., Ohmura, M., Sato, H., Matsuoka, S., Takubo, K., Ito, K., Koh, G.Y., and Suda, T. (2004). Tie2/angiopoietin-1 signaling regulates hematopoietic stem cell quiescence in the bone marrow niche. Cell *118*, 149–161.

Balazs, A.B., Fabian, A.J., Esmon, C.T., and Mulligan, R.C. (2006). Endothelial protein C receptor (CD201) explicitly identifies hematopoietic stem cells in murine bone marrow. Blood *107*, 2317–2321.

Beerman, I., Bhattacharya, D., Zandi, S., Sigvardsson, M., Weissman, I.L., Bryder, D., and Rossi, D.J. (2010). Functionally distinct hematopoietic stem cells modulate hematopoietic lineage potential during aging by a mechanism of clonal expansion. Proc. Natl. Acad. Sci. USA *107*, 5465–5470.

Benz, C., Copley, M.R., Kent, D.G., Wohrer, S., Cortes, A., Aghaeepour, N., Ma, E., Mader, H., Rowe, K., Day, C., et al. (2012). Hematopoietic stem cell subtypes expand differentially during development and display distinct lymphopoietic programs. Cell Stem Cell *10*, 273–283.

Christensen, J.L., Wright, D.E., Wagers, A.J., and Weissman, I.L. (2004). Circulation and chemotaxis of fetal hematopoietic stem cells. PLoS Biol. *2*, E75.

Cornwell, R.D., Gollahon, K.A., and Hickstein, D.D. (1993). Description of the leukocyte function-associated antigen 1 (LFA-1 or CD11a) promoter. Proc. Natl. Acad. Sci. USA *90*, 4221–4225.

Davis, D.M., Chiu, I., Fassett, M., Cohen, G.B., Mandelboim, O., and Strominger, J.L. (1999). The human natural killer cell immune synapse. Proc. Natl. Acad. Sci. USA *96*, 15062–15067.

Grakoui, A., Bromley, S.K., Sumen, C., Davis, M.M., Shaw, A.S., Allen, P.M., and Dustin, M.L. (1999). The immunological synapse: a molecular machine controlling T cell activation. Science *285*, 221–227.

Inlay, M.A., Serwold, T., Mosley, A., Fathman, J.W., Dimov, I.K., Seita, J., and Weissman, I.L. (2014). Identification of Multipotent Progenitors that Emerge Prior to Hematopoietic Stem Cells in Embryonic Development. Stem Cell Rev. *2*, 457–472.

Karlsson, G., Rörby, E., Pina, C., Soneji, S., Reckzeh, K., Miharada, K., Karlsson, C., Guo, Y., Fugazza, C., Gupta, R., et al. (2013). The tetraspanin CD9 affords high-purity capture of all murine hematopoietic stem cells. Cell Reports *4*, 642–648.

Kiel, M.J., Yilmaz, O.H., Iwashita, T., Yilmaz, O.H., Terhorst, C., and Morrison, S.J. (2005). SLAM family receptors distinguish hematopoietic stem and progenitor cells and reveal endothelial niches for stem cells. Cell *121*, 1109–1121.

Lu, R., Neff, N.F., Quake, S.R., and Weissman, I.L. (2011). Tracking single hematopoietic stem cells in vivo using high-throughput sequencing in conjunction with viral genetic barcoding. Nat. Biotechnol. *29*, 928–933.

Morrison, S.J., Wright, D.E., and Weissman, I.L. (1997). Cyclophosphamide/granulocyte colony-stimulating factor induces hematopoietic stem cells to proliferate prior to mobilization. Proc. Natl. Acad. Sci. USA *94*, 1908–1913.

Pruijt, J.F., van Kooyk, Y., Figdor, C.G., Lindley, I.J., Willemze, R., and Fibbe, W.E. (1998). Anti-LFA-1 blocking antibodies prevent mobilization of hematopoietic progenitor cells induced by interleukin-8. Blood *91*, 4099–4105.

Rossi, D.J., Bryder, D., Zahn, J.M., Ahlenius, H., Sonu, R., Wagers, A.J., and Weissman, I.L. (2005). Cell intrinsic alterations underlie hematopoietic stem cell aging. Proc. Natl. Acad. Sci. USA *102*, 9194–9199.

Seita, J., and Weissman, I.L. (2010). Hematopoietic stem cell: self-renewal versus differentiation. Wiley Interdiscip. Rev. Syst. Biol. Med. *2*, 640–653.

Spangrude, G.J., Heimfeld, S., and Weissman, I.L. (1988). Purification and characterization of mouse hematopoietic stem cells. Science *241*, 58–62.

Van Epps, D.E., Potter, J., Vachula, M., Smith, C.W., and Anderson, D.C. (1989). Suppression of human lymphocyte chemotaxis and transendothelial migration by anti-LFA-1 antibody. J. Immunol. *143*, 3207–3210.

Wagers, A.J., Allsopp, R.C., and Weissman, I.L. (2002). Changes in integrin expression are associated with altered homing properties of Lin(-/lo)Thy1.1(lo)Sca-1(+)c-kit(+) hematopoietic stem cells following mobilization by cyclophosphamide/granulocyte colony-stimulating factor. Exp. Hematol. *30*, 176–185.

Weitz-Schmidt, G., Welzenbach, K., Brinkmann, V., Kamata, T., Kallen, J., Bruns, C., Cottens, S., Takada, Y., and Hommel, U. (2001). Statins selectively inhibit leukocyte function antigen-1 by binding to a novel regulatory integrin site. Nat. Med. *7*, 687–692.

Wright, D.E., Cheshier, S.H., Wagers, A.J., Randall, T.D., Christensen, J.L., and Weissman, I.L. (2001). Cyclophosphamide/granulocyte colony-stimulating factor causes selective mobilization of bone marrow hematopoietic stem cells into the blood after M phase of the cell cycle. Blood *97*, 2278–2285.

Stem Cell Reports, Volume *3*

Supplemental Information

Upregulation of CD11A on Hematopoietic Stem Cells Denotes the Loss of Long-Term Reconstitution Potential

John W. Fathman, Nathaniel B. Fernhoff, Jun Seita, Connie Chao, Vanessa Scarfone, Irving L. Weissman, and Matthew A. Inlay

Supplemental Figures and Legends

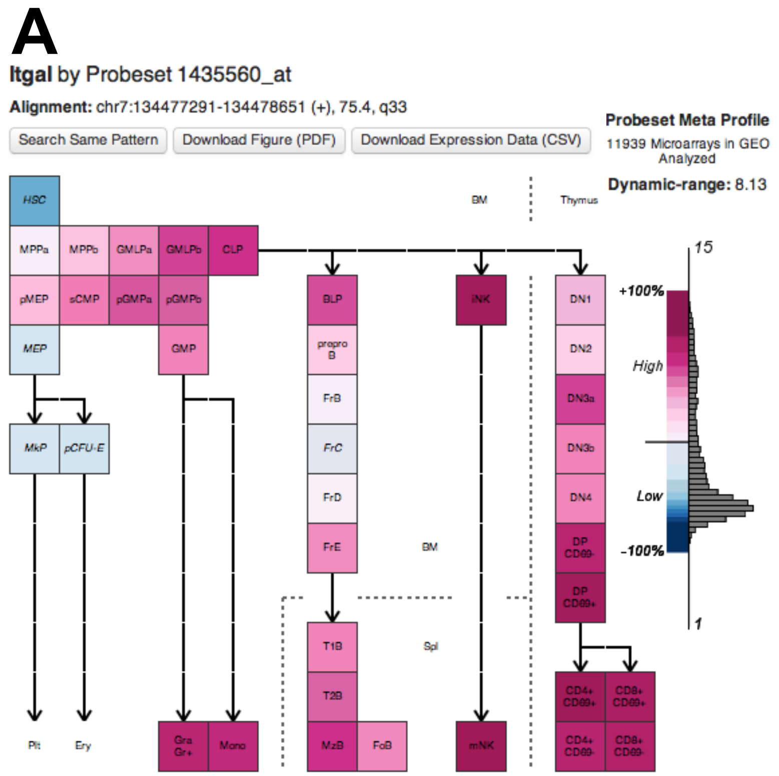

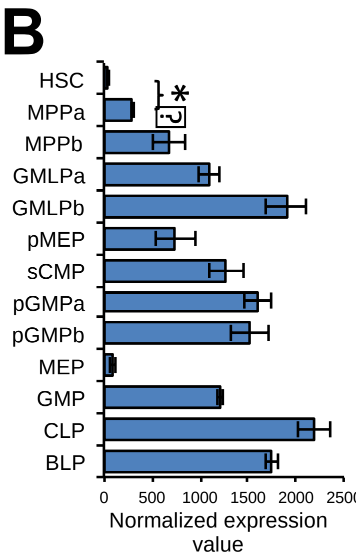

Figure S1. CD11A expression is downregulated in murine HSCs compared downstream progenitors (related to Figure 1). (**A**) Screenshot of a heat-map of CD11A expression across a panel of 39 hematopoietic populations (Seita et al., 2012). Normalized expression of a CD11A probeset (1435560_at) on Mouse 430 2.0 microarrays. Intensity of color indicates high (magenta) or low (blue) expression, as measured by percentile across a panel of nearly 11,939 microarrays of different mouse populations (right). Screenshot is taken from the Gene Expression Commons (gexc.stanford.edu). (**B**) Bar graph of CD11A microarray expression of HSCs and downstream progenitors. Normalized unlogged expression of the average CD11A expression among each population is shown. HSCs are from 4 microarrays, all others are from 3 arrays. Error bars are standard deviation. * indicates p value < 0.001 between HSC and MPPa (unpaired T-test). Please see supplemental experimental procedures for definitions of all populations. (**C**) Heat-map of cell cycle gene expression of 30 genes involved in cell cycle regulation (GO category "cell cycle") among microarrays of CD11A$^-$ and CD11A$^+$ HSC subfractions, as well as 11 downstream progenitor populations. Each row shows the relative expression of one cell cycle gene, with the intensity of colors corresponding to the percentile of expression.

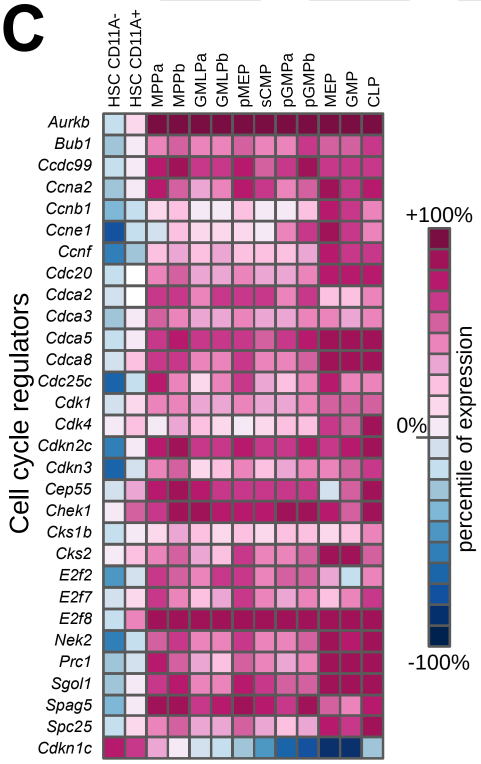

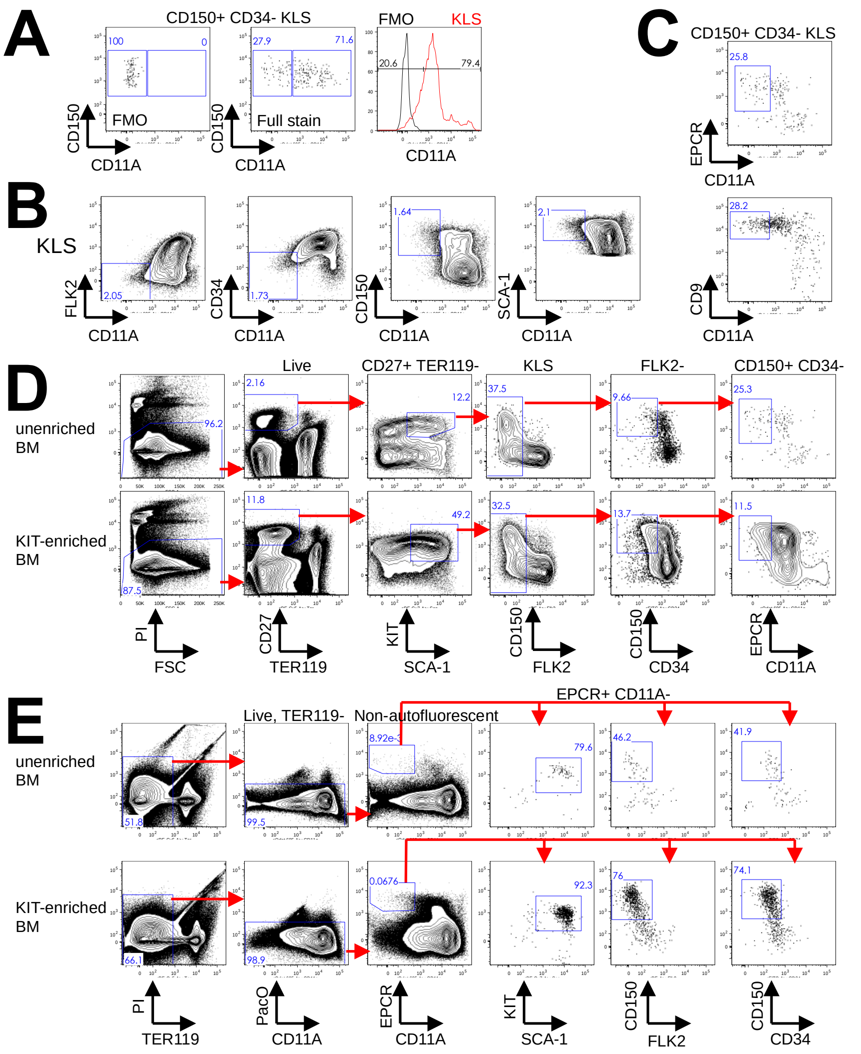

Figure S2. Guide to gating HSCs using CD11A expression (related to Figure 2). (**A**) FMO vs CD11A stain of BM HSCs. The histogram on the right shows the FMO (black) vs CD11A expression on KLS cells (red). (**B**) Comparison of CD11A to other HSC markers. CD11A (x-axis) versus other HSC markers (y-axis) of KLS cells is shown. (**C**) CD11A versus EPCR and CD9 on HSCs ($CD150^{+}$ $CD34^{-}$ KLS). Please note that the cutoff for $CD11a^{-}$ and $CD11A^{+}$ fractions is identical for all plots from S2A, S2B, and S2C. (**D**) CD11A/ EPCR expression on HSCs from unenriched (top row) versus KIT-enriched BM. The full gating strategy is shown. (**E**) EPCR and CD11A alone highly enrich for HSCs. Unenriched (top row) and KIT-enriched (bottom row) BM showing the purity of HSCs ($CD150^{+}$ $FLK2^{-}$ $CD34^{-}$ KLS) in the $EPCR^{+}$ $CD11A^{-}$ fraction. It is critical to gate out dead cells (PI^{+}), red blood cells ($TER119^{+}$) and autofluorescent cells ($PacO^{+}$), which have non-specific fluorescence in the EPCR/CD11A plot. In this case, the Pacific Orange (PacO) channel was empty and serves as a way to detect autofluorescent cells. Please note that the plots in S2D and S2E are from the same stain and only the order of gates is changed.

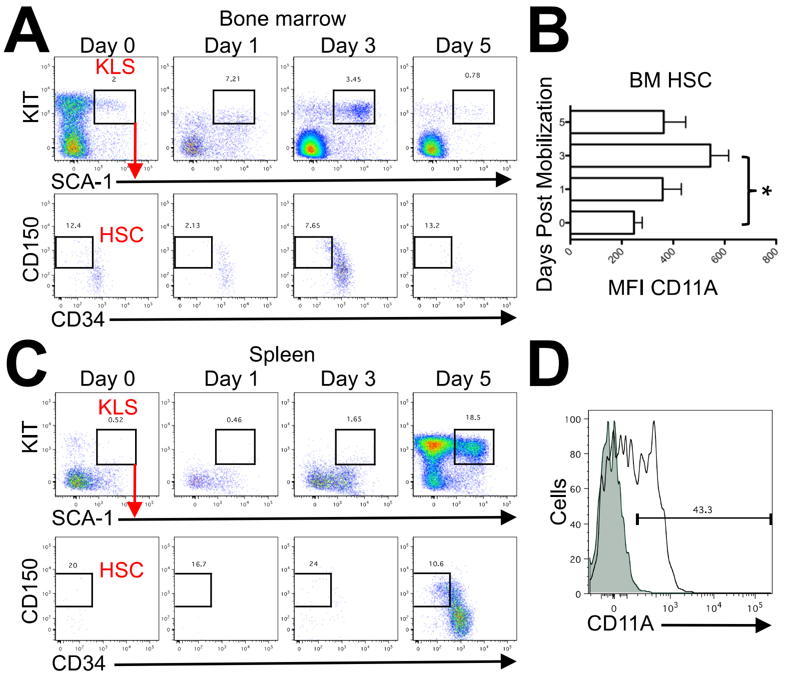

Figure S3. CD11A is upregulated on mobilized HSCs in the BM and spleen (related to Figure 3). Mobilized HSCs use these adhesion molecules to traffic from the bone marrow to the blood and subsequently the spleen. In G-CSF mobilization protocols, mice are initially injected intra-peritoneally with cyclophosphamide, which integrates into DNA and causes dividing cells to apoptose. Then on successive days sub-cutaneous injections of human G-CSF are administered. (**A**) Bone marrow of G-CSF mobilized mice were analyzed at indicated time points for expression of CD11A on HSCs. The plots in the top row were gated on Lin$^-$ cells, and the bottow row on Lin$^-$KIT$^+$SCA-1$^+$ (KLS) cells. Gates and percentages of KLS (top row) and HSCs (bottom row) are shown. Typically, by day 1 after cyclophosphamide treatment, the majority of hematopoietic cells have died, except quiescent HSCs. By day 3, HSCs become activated by G-CSF, causing them to divide and differentiate in the bone marrow. (**B**) Mean fluorescence intensity (MFI) of CD11A expression on HSCs at different time points ($p<0.0008$). (**C**) Over the following days HSCs migrate out of the bone marrow and can be found in the blood and spleen. Spleens of G-CSF mobilized mice were analyzed at indicated time points for expression of CD11A on their HSCs. Gates are the same as in Fig. 4A. (**D**) Expression of CD11A on day 5 HSCs in the spleen of mobilized mice (grey: FMO, Black line: Day 5 spleen HSCs).

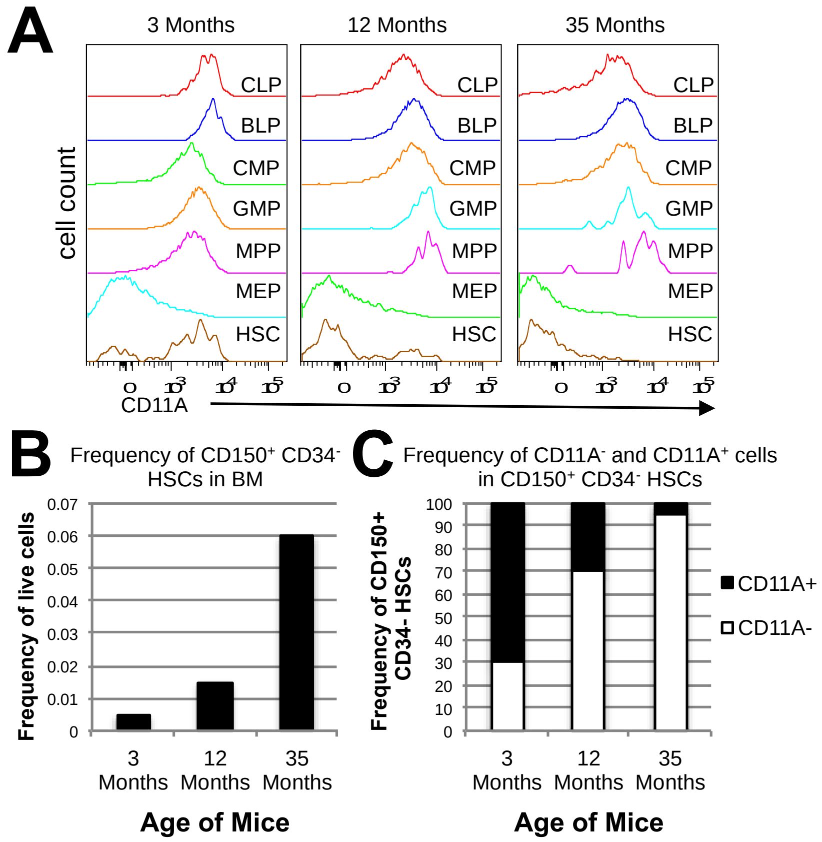

Figure S4. CD11A expression on aged HSCs (related to Figure 4). (**A**) CD11A expression profile of HSCs and downstream hematopoietic progenitors from young adult (3 month, left column), middle-aged (12 months, middle column), and old (35 months, right column) mice. (**B**) Frequency of HSCs in young, middle-aged, and old mice. (**C**) Frequency of CD11A$^-$ (white) and CD11A$^+$ (black) subfractions of HSCs in young, middle-aged, and old mice. Nearly all HSCs in old mice are CD11A$^-$.

Supplemental Experimental Procedures

Marker definitions of stem and progenitor populations used in microarray analyses

All populations used in microarray analyses are shown below and are as previously described (Seita et al., 2012). Lin = Lineage cocktail (CD3, CD4, CD8, B220, GR1, MAC1, TER119). CD11A$^-$ and CD11A$^+$ HSC populations were submitted to GEO in experiment GSE60597. Microarrays of all other populations have been previously published and submitted to GEO (GSE34723). Accession numbers for individual arrays are listed with each population in brackets.

CD11A$^-$ HSC (CD11A$^-$ Hematopoietic Stem Cell)
[GSM1483039, -40, -41]
CD27$^+$ Lin$^-$ KIT$^+$ SCA-1$^+$ FLK2$^-$ CD150$^+$ CD34$^-$ EPCR$^+$ CD11A$^-$

CD11A$^+$ HSC (CD11A$^+$ Hematopoietic Stem Cell)
[GSM1483042, -43]
CD27$^+$ Lin$^-$ KIT$^+$ SCA-1$^+$ FLK2$^-$ CD150$^+$ CD34$^-$ EPCR$^+$ CD11A$^+$

HSC (Hematopoietic stem cell, used in Figure S1A, S1B)
[GSM854035, -36, -37, -38]
CD34$^-$ FLK2$^-$ SCA-1$^+$ KIT$^+$ Lin$^-$

MPPa (Multi-potent Progenitor subset A)
[GSM854039, -40, -41]
CD34$^+$ CD150$^+$ TIE2$^+$ VCAM1$^+$ SCA-1$^+$ KIT$^+$ IL7Rα^- Lin$^-$

MPPb (Multi-potent Progenitor subset B)
[GSM854042, -43, -44]
CD34$^+$ CD150$^-$ TIE2$^+$ VCAM1$^+$ SCA-1$^+$ KIT$^+$ IL7Rα^- Lin$^-$

GMLPa (Granulo/Macrophage/Lymphoid Progenitor subset A)
[GSM854045, -46, -47]
CD34$^+$ CD150$^-$ TIE2$^-$ VCAM1$^+$ SCA-1$^+$ KIT$^+$ IL7Rα^- Lin$^-$

GMLPb (Granulo/Macrophage/Lymphoid Progenitor subset B)
[GSM854048, -49, -50]
CD34$^+$ CD150$^-$ TIE2$^-$ VCAM1$^-$ SCA-1$^+$ KIT$^+$ IL7Rα^- Lin$^-$

pMEP (pre-Megakaryocyte/Erythrocyte Progenitor)
[GSM854051, -52, -53]
CD34$^+$ FcγRII/III^{low} CD150$^+$ TIE2$^+$ VCAM1$^+$ SCA-1$^-$ KIT$^+$ IL7Rα^- Lin$^-$

sCMP (Strict Common Myeloid Progenitor)
[GSM854054, -55, -56]
CD34$^+$ FcγRII/III^{low} CD150$^-$ TIE2$^+$ VCAM1$^+$ SCA-1$^-$ KIT$^+$ IL7Rα^- Lin$^-$

pGMPa (pre-Granulocyte/Macrophage Progenitor subset A)
[GSM854057, -58, -59]
CD34$^+$ FcγRII/III^{low} CD150$^-$ TIE2$^-$ VCAM1$^+$ SCA-1$^-$ KIT$^+$ IL7Rα^- Lin$^-$

pGMPb (pre-Granulocyte/Macrophage Progenitor subset B)
[GSM854060, -61, -62]
CD34$^+$ FcγRII/III^{low} CD150$^-$ TIE2$^-$ VCAM1$^-$ SCA-1$^-$ KIT$^+$ IL7Rα^- Lin$^-$

MEP (Megakaryocyte/Erythrocyte Progenitor)
[GSM854063, -64, -65]
$CD34^-$ $Fc\gamma RII/III^-$ $SCA\text{-}1^-$ KIT^+ $IL7R\alpha^-$ Lin^-

GMP (Granulocyte/Macrophage Progenitor)
[GSM854066, -67, -68]
$CD34^+$ $Fc\gamma RII/III^+$ $SCA\text{-}1^-$ KIT^+ $IL7R\alpha^-$ Lin^-

CLP (Common Lymphoid Progenitor ($LY6D^-$))
[GSM507378, -79, -80]
Lin^- $CD11C^-$ $B220^-$ $CD27^+$ KIT^{int} $FLK2^+$ $IL7R\alpha^+$ $LY6D^-$

BLP (Earliest B-Lymphoid Progenitor)
[GSM854073, -74, -75]
Lin^- $CD11C^-$ $B220^-$ $CD27^+$ KIT^{int} $FLK2^+$ $IL7R\alpha^+$ $LY6D^+$

Microarray analysis
Microarrays (Affymetrix Mouse 430 2.0) of $CD11A^-$ and $CD11A^+$ subpopulations were generated as described (Seita et al., 2012). Microarrays were normalized to a panel of approximately 12,000 public microarrays, and the heat-maps indicate percentile of expression compared to the 12,000 arrays. Normalization and gene expression analyses were performed using the Gene Expression Commons (GEXC, gexc.stanford.edu). Microarray data included in this experiment can be accessed and analyzed on the GEXC under model 1007 (gexc.stanford.edu/model/1007).

Mobilization
Mice were injected intra-peritoneally with cyclo-phosphamide (200 mg/kg) (Sigma) and then on successive days with human G-CSF (Neupogen, 250 μg/kg) per day administered as a single daily sub-cutaneous injection. The day of cyclo-phosphamide treatment was considered day −1 and the first day of G-CSF treatment was counted as day 0. For example, mice sacrificed on day 3 of the mobilization protocol were sacrificed on the day after the third G-CSF injection

Liquid cultures
Single cells from $CD11A^-$ and $CD11A^+$ HSC subpopulations were sorted directly into individual wells of a 96-well round bottom plate containing DMEM/F12 media with 10% FBS. Growth factors SCF (Stem Cell Factor), Flt3L, IL-3, TPO (Thrombopoietin), and EPO (Erythropoietin) were added at 10 ng/ml each. Colonies were scored at day 12 of culture for number of colonies, size of colonies, and lineages produced.

Supplemental Table S1. Antibodies

Antigen	Clone	Conjugate	Source
B220	RA3-6B2	FITC	eBioscience
CD3	2C11	Alexa488	Weissman
	2C11	Alexa680	Weissman
	2C11	Pacific Blue	Weissman
	2C11	Pacific Orange	Weissman
CD4	GK1.5	Pacific Blue	Weissman
CD8	53.6.7	Pacific Blue	Weissman
CD9	MZ3	PE	Biolegend
CD11A (LFA-1)	M17/4	biotin	eBioscience
	M17/4	PE	Biolegend
CD11B (MAC1)	M1/70	PECy5	eBioscience
	M1/70	Pacific Blue	Weissman
	M1/70	Pacific Orange	Weissman
CD11C	N418	PECy5.5	eBioscience
	N418	APCCy7	eBioscience
CD16/32 (FcγRII/III)	93	APCCy7	Biolegend
CD19	1D3	PECy5.5	eBioscience
CD34	RAM34	FITC	eBioscience
CD45.1 (Ly5.1)	A20.1.7	Alexa680	Weissman
CD45.2 (Ly5.2)	AL1-4A2	PE	Weissman
CD117 (KIT)	2B8	APCCy7	eBioscience
CD127 (IL7Rα)	A7R34	PECy5	eBioscience
	A7R34	Pacific Blue	Weissman
	A7R34	biotin	eBioscience
CD135 (FLK2)	A2F10	PE	eBioscience
	A2F10	PECy5	eBioscience
	A2F10	biotin	eBioscience
CD150 (SLAMF1)	TC15-12F12.2	Alexa647	Biolegend
	TC15-12F12.2	PECy7	Biolegend
CD201 (EPCR)	eBio1560	PerCP-eFluor710	eBioscience
CD335 (NKp46)	29A1.4	FITC	eBioscience
	29A1.4	APC	eBioscience
GR1	8C5	Pacific Blue	Weissman
	8C5	Pacific Orange	Weissman
	A7R34	Pacific Blue	Weissman
	A7R34	biotin	eBioscience
Ki-67	16A8	PE	Biolegend
LY6D	49H4.3	Pacific Orange	Weissman
NK1.1	PK136	PECy5	eBioscience
	PK136	PECy7	eBioscience
SCA-1	D7	APC	eBioscience
	E13-161-7	Alexa680	Weissman
TER119	TER119	Pacific Orange	Weissman
	TER119	PECy5	eBioscience
Streptavidin		Qdot605	Invitrogen

"Weissman" denotes conjugations generated in the Weissman lab.

Supplemental References

Seita, J., Sahoo, D., Rossi, D.J., Bhattacharya, D., Serwold, T., Inlay, M.A., Ehrlich, L.I., Fathman, J.W., Dill, D.L., and Weissman, I.L. (2012). Gene Expression Commons: an open platform for absolute gene expression profiling. PLoS One *7*, e40321.
